# Bacteriocins: Properties and potential use as antimicrobials

**DOI:** 10.1002/jcla.24093

**Published:** 2021-12-01

**Authors:** Atieh Darbandi, Arezoo Asadi, Marzieh Mahdizade Ari, Elnaz Ohadi, Malihe Talebi, Masoume Halaj Zadeh, Amir Darb Emamie, Roya Ghanavati, Maryam Kakanj

**Affiliations:** ^1^ Department of Microbiology School of Medicine Iran University of Medical Sciences Tehran Iran; ^2^ Microbial Biotechnology Research Centre Iran University of Medical Sciences Tehran Iran; ^3^ Department of Pathobiology School of Public Health Tehran University of Medical Sciences Tehran Iran; ^4^ Behbahan Faculty of Medical Sciences Behbahan Iran; ^5^ Food and Drug Laboratory Research Center Food and Drug Administration MOH&ME Tehran Iran

**Keywords:** bacteriocin, foodborne pathogens, lactic acid bacteria

## Abstract

A variety of bacteriocins originate from lactic acid bacteria, which have recently been modified by scientists. Many strains of lactic acid bacteria related to food groups could produce bacteriocins or antibacterial proteins highly effective against foodborne pathogens such as *Staphylococcus aureus*, *Pseudomonas fluorescens*, *P*. *aeruginosa*, *Salmonella typhi*, *Shigella flexneri*, *Listeria monocytogenes*, *Escherichia coli* O157:H7, and *Clostridium botulinum*. A wide range of bacteria belonging primarily to the genera *Bifidobacterium* and *Lactobacillus* have been characterized with different health‐promoting attributes. Extensive studies and in‐depth understanding of these antimicrobials mechanisms of action could enable scientists to determine their production in specific probiotic lactic acid bacteria, as they are potentially crucial for the final preservation of functional foods or for medicinal applications. In this review study, the structure, classification, mode of operation, safety, and antibacterial properties of bacteriocins as well as their effect on foodborne pathogens and antibiotic‐resistant bacteria were extensively studied.

## Graphical abstract

Antimicrobial potential of bacteiocins in food preservation.

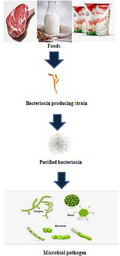

## 1 INTRODUCTION

There are a variety of antimicrobial substances produced by living organisms as part of their immune reactions or defense mechanisms, which may enhance their ability to compete for space and resources. Especially, the production of antimicrobial substances such as hydrogen peroxide, fatty acids, organic acids, ethanol, lytic enzymes, antibiotics, and bacteriocins by living organisms is well evidence of their microbial antagonism.^1^, ^2^, ^3^ Bacteriocins are a very heterogeneous class of bactericidal peptides or proteins produced by bacteria and archaea.^3^ The vast majority of all bacterial species appear to be able to produce at least one bacteriocin, most of which are unknown.^4^ Bacteriocins exhibit antibacterial activity and a specific immunity mechanism toward strains closely related to the producer bacteria.^3^, ^4^ This immunity is usually associated with a specific protein encoded in the bacterial genome (bacteriocin operon).^5^, ^6^ Almost all bacteriocins are small cationic molecules with hydrophobic or amphiphilic characteristics, which exhibit a narrow to broad inhibitory activity against closely related and non‐related species.^7^, ^8^ Bacteriocins may be secreted by both Gram‐positive and Gram‐negative bacteria. In particular, among Gram‐positive bacteria, bacteriocins derived from lactic acid bacteria are of great interest (LAB).^9^ Lactic acid bacteria are a miscellaneous group of acid‐tolerant, facultative anaerobes, and fermentative organisms that possess generally regarded as safe (GRAS) and qualified presumption of safety (QPS) status. Therefore, their bacteriocins are regarded as safe by the USFDA (the U.S. Food and Drug Administration).^2^, ^5^, ^10^ LAB‐secreted bacteriocins are heat‐stable, proteases‐sensitive, ribosomally synthesized, and antimicrobial peptides, which either undergo or do not undergo enzymatic post‐translational modification process.^2^, ^4^, ^6^, ^9^, ^11^ They are very diverse in terms of length, genetic origin, biochemical features, molecular weight (MW), cellular receptors, and interaction with the immune system.^5^, ^9^, ^12^ So far, many bacteriocins have been widely identified with various modes of action as follows: inducing cytoplasmic membrane permeability (by pore formation), inhibiting cell wall biosynthesis, and interfering with metabolic pathways.^1^, ^6^ Membrane pore formation as a common inhibitory mechanism, high inhibitory activity with low concentrations in the nanomolar range, and relatively narrow‐spectrum antimicrobial activity are the characteristics of many LAB‐derived bacteriocins.^1^ During the last decade, LAB bacteriocins have received a great deal of attention due to their high potential, especially as natural food preservatives and novel therapeutic antibiotics, which have made them attractive for various applications.^13^ In particular, numerous studies have been published in recent years, suggesting that bacteriocins may be used as an alternative to antibacterial agents in the prevention or treatment of bacterial infections.^10^ Despite the long history of bacteriocins usage, there is no report about the development of bacterial resistance to bacteriocins. Some possible reasons may be as follows: having a fast pore‐formation mechanism, being easily degraded by proteolytic enzymes, and having a shorter biological half‐life in the human body or in natural environments; these factors minimize the possibility of interaction between bacteriocins and bacteria, as a starting point in the development of antibiotic resistance.^13^ The most significant property that further enhances bacteriocins functional potential compared with conventional antibiotics is stability in a wide range of physicochemical conditions. In addition, due to their primary metabolite nature, bacteriocins also have other features such as genetic amenability through bioengineering to increase their activity/specificity against target bacterial pathogens and antibiotic‐resistant strains.^13^ Lastly, bacteriocins modulate the host immune responses (as signaling peptides), and they have no destructive effect on commensal bacteria and low/no cytotoxicity against eukaryotic cells (more specifically to target cells).^3^, ^14^ Comparison of bacteriocins and antibiotics in terms of mode of action and physicochemical properties, especially the mechanism of resistance development (used by bacteria), provides evidence of the need for alternative antimicrobial compounds to combat bacterial infections. Bacteriocins have the potential to be used as ideal candidates.^10^

In recent years, several studies have shown the protective effects of LAB bacteriocins on the gastrointestinal tract via eliminating pathogens or supporting the gut from bacterial colonization.^2^ They have also demonstrated the inhibitory ability of bacteriocins against intestinal pathogens such as *Listeria monocytogenes*, *Salmonella enteritidis*, *Clostridium difficile*, *Staphylococcus aureus*, and vancomycin‐resistant enterococci (VRE).^14^ Moreover, in clinical applications, some bacteriocins have been shown to be highly effective in treating infections, especially those caused by multidrug‐resistant (MDR) strains.^13^ Given the harmful effect of antibiotics on intestinal microbiota and the importance of gastrointestinal infections in human health, it seems that LAB‐derived bacteriocins could be considered as a promising antibiotic in the treatment of intestinal infections.^2^ This research aimed to review the literature regarding the properties of bacteriocins as a substitute for antibiotic therapy, their antibacterial effects on foodborne pathogens, their impact on antibiotic‐resistant bacteria, and the identification and extraction of new bacteriocins against intestinal pathogens.

## 2 THE BIOLOGY OF BACTERIOCINS

Bacteriocins were first identified almost 100 years ago as cationic, ribosomally synthesized, antimicrobial peptides, which act through forming pore and disrupting target cell membrane integrity, eventually leading to cell death. Lactic acid bacteria (LAB), in particular, are known to produce a variety of bacteriocins with characteristics that make them ideal for use in inactivating pathogens, including their broad antimicrobial activity spectrum, pH‐ and heat‐tolerance, and non‐toxic nature. In addition, LAB bacteriocins are sensitive to digestive proteases such as pancreatic complex, host proteases, trypsin, and chymotrypsin; thus, they do not adversely affect the gut microbiota.^15^ According to Klaenhammer,^16^ 99% of all bacteria probably produce at least one bacteriocin, most of which have not yet been identified because few researchers have looked for them. Most bacteriocins have two main characteristics that distinguish them from traditional antibiotics: Bacteriocins are ribosomally synthesized and have a relatively narrow‐spectrum antimicrobial activity.^17^ Bacteriocins are a family of proteins that could be classified into two classes based on their size, microbial target, mode of action, release, and immunity mechanisms, including bacteriocins of Gram‐negative and Gram‐positive bacteria.^18^ Bacteriocins may represent a good potential for use as substitutes for antibiotics with no side effects. Bacteriocins may have different modes of action, including inhibition of cell wall synthesis, inhibition through DNase and RNase activities, and more commonly, formation of pores in the target cell membrane. Lactic acid bacteria are ideal candidates for bacteriocin production in order to biocontrol of some bacterial species as they have a long history of safe use in the food industry.^19^ Inoculation of food with bacteriocin‐producing strains and addition of purified or semi‐purified bacteriocins, which promote either a bactericidal effect with/without cell lysis or a bacteriostatic effect inhibiting cell growth, are the most common approaches to using bacteriocins for food biopreservation.^20^ Most LAB bacteriocins, especially those inhibiting Gram‐positive bacteria, exert their antimicrobial effects by targeting bacterial cell envelope.^21^

## 3 WHAT ARE THE SOURCES OF BACTERIOCINS?

Antimicrobial peptides are natural and unique antibiotic substances produced by different microorganisms, mammals, plants, and insects.^22^, ^23^ Bacteriocin‐producing bacteria could be extracted from various sources such as water, soil, animal intestines, and food products, which are defined as unconventional sources, while the human gastrointestinal tract is considered as the conventional source of bacteriocin producers. Understanding more about unconventional sources, especially food products^24^, ^25^ and animals,^26^ has become interesting for researchers in recent years. Bacteriocin‐producing bacteria in the GIT include *Lactobacillus*, *Enterococcus*, *Streptococcus*, and *Staphylococcus*.^24^ These bacteria could help the skin serve as the first line of body's defense against pathogens. In addition to inhibiting the growth of other bacteria, these bacteriocins as mediator molecules are able to help the immune system.^27^, ^28^, ^29^, ^30^

## 4 UNCONVENTIONAL SOURCES OF BACTERIA THAT PRODUCE BACTERIOCIN

4.1 In **dairy** products, the most common LAB strains include *Lactococcus lactis* and *L*. *plantarum* in camel and goat milk, *L*. *plantarum* in cheese, *L*. *kefiranofaciens* in kefir, and *S*. *thermophilus*, and *L*. *brevis* LSe in other dairy products. In dairies such as milk of different origins, *L*. *brevis* produces bacteriocin with antibacterial activity. *Brevibacillus brevis* and *Bifidobacterium lacti*s are the most common LAB in raw milk, while *L*. *acidophilus* is the most common LAB in yogurt and fermented soy products.^31^ Generally, *Lactococcus*, *Lactobacillus*, and *Streptococcus* are the predominant probiotic bacteria in milk products.^32^
4.2 *L*. *plantarum* subsp. *plantarum*, *Pediococcus pentosaceus*, *L*. *brevis*, *L*. *curvatus*, and *L*. *fermentum* are the most widely used bacteriocin‐producing bacteria in fermented raw‐meat products (like sausage); *E*. *faecium* UAM1 is the most common LAB in cooked meat products, while *L*. *plantarum*, *L*. *brevis*, and *P*. *pentosaceus* strains are common in other fermented meat products.^25^, ^33^ These LAB strains exhibit inhibitory activity on *L*. *monocytogenes*, *Aeromonas hydrophila*, and *S*. *aureus* and prevent the growth of these foodborne pathogens.^34^, ^35^, ^36^
4.3 The most common LAB strains in **seafood** are *L*. *brevis* LAP2, *L*. *plantarum*, and *E*. *faecium* HL7.^26^ It has been proved that LAB strains in fish and shrimp show antibacterial activity against foodborne pathogens and other bacteria.^37^ *A*. *hydrophila* as a common freshwater fish pathogen could be significantly inhibited by probiotic activity of bacteriocin‐producing pathogens in fish gut.^38^ In a study, LAB isolated from the intestine of a fish named *Mugil cephalus* showed inhibitory function against fish and even human pathogens.^39^
4.4 *L*. *brevis*, *Lactiplantibacillus pentosus*, *Lacticaseibacillus paracasei*. *Lacticaseibacillus plantarum*, *Limosilactobacillus fermentum*, *Lactobacillus*, *Weissella*, *Pediococcus*, and *E*. *durans* are the most important LAB in **fruit and vegetables** (raw fruits and vegetables, carrots, pineapple, and different fruits).^32^

Some bacteria residing in the sea or ocean, such as *Aeromonas*, *Alteromonas*, and *Vibrio*, are capable of exhibiting a strong bactericidal effect on drug‐resistant bacteria and boosting the immune system by producing bacteriocin.^30^

## 5 BACTERIOCINS PRODUCED BY LACTIC ACID BACTERIA

Bacteriocins, as a group of diverse antimicrobial peptides, are known as an inhibitory agent against closely related organisms such as Gram‐positive and Gram‐negative bacteria, they are produced by various bacterial species, specifically lactic acid bacteria (LAB).^5^, ^40^, ^41^ It has also been well documented that LAB strains are commonly involved in a broad spectrum of ecological niches and include various genera, such as *Pediococcus*, *Leuconostoc*, *Weissella*, *Lactococcus*, *Lactobacillus*, *Streptococcus*, *Enterococcus*, *Carnobacterium*, *Propionibacterium*, *Aerococcus*, *Tetragenococcus*, *Oenococcus*, *and Bifidobacterium*.^40^, ^42^ Bacteriocins could be categorized into three major classes. Class I bacteriocins are small post‐translationally modified peptides, which are/could be further subdivided into Class Ia (lantibiotics), Class Ib (labyrinthopeptins), and Class Ic (sanctibiotics).^43^ Nisin is a Class I bacteriocin. Class II bacteriocins are small, heat‐stable, non‐modified peptides described as unmodified bacteriocins, which could be further subdivided into Class IIa (pediocin‐like bacteriocins), Class IIb (two‐peptide unmodified bacteriocins), Class IIc (circular bacteriocins), and Class IId (unmodified, linear, non‐pediocin‐like bacteriocins). In addition, Class III bacteriocins as larger peptides (>10 kDa, heat‐labile) are subdivided into two subclasses, namely IIIa (lysostaphin and enterolysin A) or bacteriolysins and IIIb.^42^, ^44^, ^45^ Bacteriocins are commonly named based on the genus or species of producer strain. For instance, lacticin and nisin are produced by *Lactococcus* spp., enterocin by *Enterococcus* spp., pediocin by *Pediococcus* spp., leucocin by *Leuconostoc* spp., etc.^40^

## 6 BIOSYNTHESIS AND POTENTIAL APPLICATION OF LAB BACTERIOCINS

There are some factors affecting the production of bacteriocins, such as culture conditions and the type of microbial strain. Bacteriocins are post‐translationally modified and ribosomally synthesized peptides that are initially produced as inactive pre‐peptides and then converted into an active form.^5^, ^46^ After transport and cleavage of produced pre‐peptides, they are converted into mature (active) bacteriocins. Depending on the type of bacteriocins, genes responsible for pre‐peptides transport and modification are located close to the bacteriocin biosynthesis gene, and genes responsible for the immunity and active bacteriocin production are broadly located in operon clusters and might be harbored in plasmid, genome, or other mobile genetic factors.^47^ In addition to inducible expression, these operons require auto‐inducer peptides to induce bacteriocin production. The regulation of expression is generally fulfilled by a two‐part regulatory system, but in some cases, this process is done by three‐element ones. The production of bacteriocins is fulfilled in the exponential growth phase by retaining a line‐to‐line connection to the biomass production.^44^

## 7 ACTIVITY OF LAB AGAINST FOODBORNE PATHOGENS

According to the FDA, the most prevalent foodborne pathogenic bacteria are as follows: *L*. *monocytogenes*, *S*. *enteritidis*, *Shigella*, *S*. *typhimurium*, pathogenic *Escherichia coli*, *Campylobacter jejuni*, *C*. *perfringes*, and *S*. *aureus*.^41^ Due to some restrictions on the use of therapeutic antibiotics in food production and processing as well as nontoxicity of treatment with LAB‐derived antimicrobial peptides, most research in recent years has been devoted to investigating these bacteriocins. Given the cleavage of bacteriocins by gastrointestinal proteases, reducing their activity level, they are generally safe and show a considerable capacity to constrain foodborne pathogens growth.^41^, ^48^ Also, considering bacteriocins ability to form pores in the cell membrane of sensitive bacteria, they are naturally bactericidal or bacteriostatic. For instance, BMP11 as a new bacteriocin produced by *L*. *crustorum* against *L*. *monocytogenes*, has been shown to destroy cell membrane integrity and increase membrane permeability.^49^ Moreover, it has been well documented that *P*. *acidilactici* QC38 is able to inhibit *L*. *monocytogenes*, *L*. *innocua*, *S*. *typhimurium*, *E*. *coli*, *V*. *cholerae* NO 01, and *V*. *cholerae* O1 *Ogawa*.^50^ The inhibitory activity of *Pediococcus* spp. (isolated from cheese) against *Listeria* species was reported by Cavicchioli et al.^51^ They indicated that bacteriocins produced by *E*. *hirae* ST57ACC and *P*. *pentosaceus* ST65ACC inhibited 100% of *L*. *monocytogenes* strains and two *L*. *innocua* strains. Nisin, produced by *L*. *lactis* subsp. *Lactis*, has been reported to inhibit the growth of Gram‐positive bacteria as well as *Clostridium* and *Bacillus* spores. Considering its antimicrobial activity against *Listeria* spp., pediocin (*P*. *acidilactici*) has been shown to mitigate the growth of spoilage microorganisms during storage in the meat industry as well as to be efficient against *L*. *monocytogenes* in beef, turkey, and sliced jambon.^44^ Similar to pediocin, the semi‐purified bacteriocin BacTN635 (produced by *L*. *plantarum* sp. TN635) has also been shown to be strongly active against spoilage microorganisms in chicken breast and beef.^52^ Furthermore, the potency of combined application of various bacteriocins, including subclass IIa bacteriocins, *L*. *fermentum ACA*‐*DC179*, and bioprotective cultures of *E*. *faecium PCD71*, in inhibiting *L*. *monocytogenes* in various meat products has been documented. In addition to enterocins as broad‐spectrum antimicrobial bacteriocins against foodborne pathogens (*Clostridium* spp. and *Listeria* spp.),^45^ purified bifidocin A has also been indicated to display a broad range of antimicrobial activity against spoilage and foodborne pathogenic bacteria, such as *S*. *aureus*, *L*. *monocytogenes*, *E*. *coli*, and some types of yeasts.^53^

## 8 ARE BACTERIOCINS THE BEST SUBSTITUTE FOR ANTIBIOTIC THERAPY?

To the best of our knowledge, antibiotics play an important role in the disease prevention and treatment in animals and humans. In addition to adverse effects of some antibiotics, the emergence of antibiotic‐resistant, MDR (multidrug‐resistant), and XDR (extensively drug‐resistant) strains has recently become a major concern.^54^, ^55^ It is estimated that by 2060, at least 20 new antibiotics are needed to overcome the problem of antibiotic resistance, while the design of new antibiotics is a time‐consuming and slow procedure.^56^ Therefore, it is necessary to develop new treatment strategies that could eliminate antibiotic‐resistant microorganisms.^57^ One of the strategies is to use antimicrobial peptides to achieve this goal. Bacteriocin is considered as a suitable antimicrobial peptide due to its thermal stability and high efficacy with nano‐molecular size.^58^, ^59^ In addition to the immune system, bacteriocins are able to affect other bacteria through competition in colonization.^21^, ^33^ Researchers are looking for anti‐pathogenic bacteriocins that are as effective as antibiotic therapy.^60^ Bacteriocins have been shown to possess advantages over antibiotics.^61^ These antimicrobial peptides are considered to provide more protection with no side effects compared to antibiotics. A study examining the differences between bacteriocins and antibiotics found that oral administration of pediocin PA‐1 had no side effects on the gastrointestinal tract, while under the same conditions, the use of antibiotics such as penicillin and tetracycline exhibited different results.^62^ Bacteriocins are synthesized ribosomally and considered as primary metabolites, while antibiotics are a type of secondary metabolites. This feature allows researchers to design novel bacteriocins with more effective capabilities using bioengineering techniques based on bacteriocins synthesis ways.^63^ Unlike antibiotic‐producing bacteria, bacteriocins are not inhibited by antimicrobial agents. The activity of bacteriocins on drug‐resistant strains, hospital‐acquired infections, respiratory tract infections, skin diseases, dental infections, vaginosis, and tuberculosis has been proved.^62^ Several antibiotics are not active against infections caused by *S*. *aureus*, enterococci, and pneumococci strains. Studies have shown that MRSA (methicillin‐resistant *S*. *aureus*) and VRE (vancomycin‐resistant enterococci) are affected by bacteriocins such as nisin A and lacticin 3147.^64^ Nisin F and mersacidin were shown in a study to inhibit *S*. *aureus* growth as a respiratory pathogen without damaging the lungs and other respiratory organs such as bronchi and trachea in animal model.^65^ Proteolytic digestion of bacteriocins in the gastrointestinal tract may affect bacteriocins activity; however, it is possible to overcome the problem by their encapsulation and insertion into liposomes.^63^ Also, the use of synergistic function of bacteriocins is economically more cost effective than producing expensive antibiotics.^63^ The inherent resistance of certain bacteria to these peptides with very strong antimicrobial activity at nanomolar concentrations is one of the most challenging aspects of bacteriocins usage. Although few studies have examined resistance to bacteriocins, some studies have shown a nearly 10‐fold increase in nisin MIC against *L*. *monocytogenes*, indicating a 10‐fold increase in resistance to nisin. Degradation of nisin by *B*. *cereus*‐synthesized nisinase leads to inactivation of this bacteriocin, while bacitracin, polymyxin, and gramicidin are resistant to these enzymes.^66^, ^67^ Unlike widespread studies on enzymes mediating extensive resistance to β‐lactam antibiotics, such as β‐lactamases and carbapenemases, resistance of bacteriocin‐degrading enzymes has not been properly investigated.^68^ Therefore, there is controversy over whether bacteriocins could be considered as alternatives to antibiotics or not. Studies results describe bacteriocins as supplements for antibiotics due to their high stability, no side effects, and potential to induce a synergistic effect.^69^ In combination with antibiotics, bacteriocins act synergistically; they not only increase the efficiency of antibiotics and prevent the emergence of antibiotic‐resistant species but also reduce the side effects of antibiotics by lowering the concentration of antibiotics needed to eliminate bacteria.^61^, ^63^, ^70^, ^71^ Nowadays, the therapeutic efficacy of bacteriocins could be enhanced by designing engineered bacteriocins; this requires biological and chemical modification methods and further investigations.^57^

## 9 IMPACT OF BACTERIOCINS ON ANTIBIOTIC RESISTANCE PROFILE OF DRUG‐RESISTANT BACTERIA

The incidence of antibiotic resistance is attributed to several factors, including overuse of antibiotics, self‐medication at home, and improper antibiotic administration. When people consume contaminated food with such drug‐resistant bacteria, they lose sensitivity to the relevant antibiotics.^72^ According to available information, many patients die due to antibiotic resistance with an annual rate of at least 700,000 cases, and this number is likely to increase by 2050. Some bacteriocins such as nisins A and F, mersacidin, mutacin 1140, lactacin 3147, and pediocin AcH/PA‐1 are active against MRSA and VRE strains.^65^, ^73^, ^74^ Brand et al.^75^ indicated that nisin F could inhibit *S*. *aureus* infection in mice and reported that the growth‐inhibition time lasted only 15 minutes. Another study by Fernández et al. (2008) demonstrated the antibacterial activity of nisin A in mastitis. They also described bacteriocins as efficient alternatives to antibiotics and showed that after two weeks, the number of bacteria in the milk of mothers with mastitis was significantly reduced and then followed by relief of symptoms.^76^ It has been shown that *Bacillus* spp. are able to reduce MRSA infection in animal model by producing mersacidin.^73^ Bacteriocin LipA is active against *Pseudomonas aeruginosa* and could eliminate resistant strains. Drug‐resistant *S*. *aureus* strains in goat milk are also affected by AS‐48 and nisin. These bacteriocins both individually and synergistically are able to eliminate *S*. *aureus*.^77^ Researchers have shown that *B*. *subtilis* KIBGE IB‐17 produces a heat‐resistant bacteriocin called BAC‐IB‐17 which is very effective against MRSA strains.^78^ Also, the effect of another bacteriocin called sonorensinon has been proven against various Gram‐positive bacteria, such as antibiotic‐resistant *S*. *aureus* biofilms and Gram‐negative bacteria.^79^ In an animal study in the field of bacteriocin design, bacteriocin peptide Ω76 was introduced as an active substance against carbapenem‐ and tigecycline‐resistant *Acinetobacter baumannii*. However, in this study, the relevant bacteriocin showed toxic effects such as nephrotoxicity, which was resolved in combination with antibiotics.^80^

## 10 IS IT POSSIBLE TO CONTROL FOODBORNE BACTERIA BY BACTERIOCINS?

Food pathogenic bacteria are present in both planktonic and biofilm forms as foodborne pathogens. In addition to being able to counteract with drug‐resistant bacteria, bacteriocins may also be used as food and dairy products preservatives.^81^ According to studies, LAB‐derived bacteriocins such as nisin, pediocin PA‐1, pediocin, mersacidin, mutacin, and lacticin are mostly used in the food processing industry as preservatives that are capable of preventing the growth of *C*. *botulinum*, *E*. *faecalis*, VRE, *L*. *monocytogenes*, *S*. *aureus*, and other foodborne pathogens.^82^, ^83^, ^84^ *L*. *monocytogenes* as the most common meat food pathogen could cause serious infections. Huang et al.^85^ showed that enterocin RM6 and enterocin AS‐48 as cyclic peptides generated by *E*. *faecalis* (found in raw‐milk) were able to affect some foodborne bacteria, especially *L*. *monocytogenes*, *B*. *cereus*, and MRSA strains. Studies have shown the effects of various bacteriocins on *L*. *monocytogenes*.^82^, ^86^, ^87^, ^88^ *B*. *cereus* is another important foodborne pathogen causing different opportunistic infections in immunocompromised and immunocompetent individuals, such as food poisoning. Lauková et al indicated that lantibiotic bacteriocins, namely, gallidermin, and nisin, can be prevent from biofilm‐forming enterococci multiplication isolated by Slovak dry fermented meat.^89^ Draper et al.^90^ showed that lacticin 3147 as a lantibiotic in combination with polymyxin B could be active against *B*. *cereus* 8079 and *B*. *cereus* 5247. There are other bacteria known as foodborne pathogens that have been extensively studied by researchers to evaluate the effect of different bacteriocins on their growth and proliferation. For example, elimination of *S*. *entrica* serovar *typhi* following the use of nisin along with β‐lactam antibiotics was proven in a study by Rishi et al.^91^ Although bacteriocins have become popular as an antimicrobial peptide in recent decades,^62^ various studies have currently shown that bacteriocins in combination with conventional antibiotics could have a broader and stronger effect; however, the role of bacteria as an antimicrobial agent in the management of infections is still controversial.

## 11 CLASSIFICATION OF BACTERIOCINS

Up to now, four classes of LAB bacteriocins have been identified: Class I, known as lantibiotics, is composed of modified bacteriocins; Class II includes heat‐stable, minimally modified bacteriocins; Class III includes larger, heat‐labile bacteriocins; and Class IV is composed of complex bacteriocins with carbohydrate or lipid moieties. Classes I and II bacteriocins have been the focus of most probiotic research.^92^ Lantibiotics are divided into three groups based on their structure and mode of action. Type A lantibiotics, such as nisin, are small (2–5 kDa) proteins with positively charged molecules, which kill bacteria via the formation of membrane pores, causing dissipation of membrane integrity and efflux of small metabolites from the sensitive cells. Mersacidin is a member of Type B lantibiotics that kill bacteria by interfering with cellular enzymatic reactions, such as cell wall synthesis.^93^ Another subgroup, such as lacticin 3147, is two‐component lantibiotics that synergistically display antimicrobial activity. It has been shown that dual activities could be distributed across two peptides: While one resembles Type B lantibiotic mersacidin that depolarizes the membrane, the other one is more similar to Type A lantibiotic nisin that acts through pore formation. Class II LAB bacteriocins are also small non‐lanthionine‐containing peptides that kill bacteria by inducing membrane permeability and the subsequent leakage of molecules from target bacteria. These bacteriocins are organized into the following subgroups: Class IIa is the largest group, including pediocin (this group is also called pediocin‐like bacteriocins), sakacin A, and leucocin A. Like Type A lantibiotics, Class IIa bacteriocins act through the formation of pores in the cytoplasmic membrane. Class IIa bacteriocins are distinguished by shared activity against *Listeria* and a conserved amino‐terminal sequence (YGNGVXaaC) that is thought to facilitate nonspecific binding to the target surface. Class IIb bacteriocins, such as lacticin F and lactococcin G, are composed of two different proteins; they form pores in the cell membrane to inhibit or kill their target bacteria. Class IIc bacteriocins are sec‐dependent, such as acidocin 1B.^94^ Class III bacteriocins are large, heat‐labile proteins such as helveticins J or lactacin B. Class IV bacteriocins such as leuconocin S and lactocin 27 require lipid or carbohydrate moieties for activity.^95^

### 11.1 Bacteriocins of Gram‐positive bacteria

Bacteriocins of Gram‐positive bacteria are as abundant as and even more diverse than those found in Gram‐negative bacteria. Bacteriocins of Gram‐positive bacteria, in general, and lantibiotics, in particular, require more genes for their production than those of Gram‐negative bacteria. The nisin gene cluster, for example, includes genes for encoding the pre‐peptide (nisA), modifying amino acids (nisB, nisC), cleavage of the leader peptide (nisP), secretion (nisT), immunity (nisI, nisFEG), and regulation of expression (nisR, nisK). These gene clusters are most often encoded on plasmids but are occasionally found on chromosomes. In several Gram‐positive bacteria, bacteriocins synthesis genes, including nisin, are located on transposons.^96^ Bacteriocins of Gram‐positive bacteria are restricted to eliminate other Gram‐positive bacteria. The range of their antimicrobial activity could vary significantly from relatively narrow spectrum, such as in lactococcins A, B, and M, which have been found to kill only *Lactococcus*, to extraordinarily broad spectrum, such as in some Type A lantibiotics including nisin A and mutacin B‐Ny266, which have been shown to kill a wide range of organisms, including *Actinomyces*, *Bacillus*, *Clostridium*, *Corynebacterium*, *Enterococcus*, *Gardnerella*, *Lactococcus*, *Listeria*, *Micrococcus*, *Mycobacterium*, *Propionibacterium*, *Streptococcus*, and *Staphylococcus*. Contrary to conventional wisdom, these particular bacteriocins are also active against a number of medically important Gram‐negative bacteria including *Campylobacter*, *Haemophilus*, *Helicobacter*, and *Neisseria* strains as well as methicillin‐resistant bacteria.^97^

#### 11.1.1 Bacteriocins of *Lactobacillus*

The growing emergence of antibiotic‐resistant bacteria in the food industry needs to be controlled with effective antimicrobials. Plantaricin is one of the most important known bacteriocins produced by *Lactobacillus*. *Lacticaseibacillus plantarum* could produce at least six different bacteriocins. Several bacteriocin‐producing strains of *L*. *plantarum* are used in the food production.^98^ Zhang et al. (2018) in their study indicated that bacteriocin J23 produced by *L*. *plantarum* J23, isolated from Chinese traditional fermented milk products, showed thermal stability at temperatures below 100°C for 30 min, pH stability in the range of 2.0 to 12.0, and strong activity against *L*. *monocytogenes*. Their study results suggest that bacteriocin J23 have potential application prospects in the food industry.^99^ Díaz et al.^100^ demonstrated that plantaricin‐producing *L*. *plantarum* strain EC52 isolated from poto poto exhibited a strong inhibitory activity against *L*. *monocytogenes* and *E*. *coli* O157:H7 in meat. Omar et al.^101^ indicated that bacteriocin‐producing *L*. *plantarum* C11 isolated from the Congolese fermented maize product poto poto exhibited inhibitory activity against *S*. *enterica*, *E*. *coli*, *S*. *aureus*, *E*. *aerogenes*, *B*. *cereus*, *E*. *faecalis*, and *L*. *monocytogenes* and could be used to improve the safety and storage stability of poto poto. Plantaricin JLA‐9 as a new bacteriocin was purified by Zhao et al.^102^ and showed broad‐spectrum antibacterial activity against Gram‐positive and Gram‐negative bacteria, especially *Bacillus* spp. This bacteriocin could prevent the growth of *B*. *cereus* spores. ST28MS and ST26MS bacteriocins produced by two different strains of *L*. *plantarum* were purified in a study from molasses and showed inhibitory activity against Gram‐negative bacteria, including *P*. *aeruginosa*, *E*. *coli*, and *A*. *baumannii*.^103^ Two bacteriocins JW3BZ and JW6BZ produced by *L*. *plantarum* (isolated from Bulgarian boza) were shown to be active against a wide range of Gram‐positive bacteria by Von Mollendorff et al.^104^ Also, plantaricin C‐11,^105^ plantaricin NA,^106^ and bacteriocin AMA‐K have been shown to have a strong anti‐*Listeria* activity and therefore may be used in food preservation in the future.^107^, ^108^ Amortegui et al.^109^ presented the characteristics of bacteriocins produced by *L*. *plantarum* LE5 and LE27 isolated from ensiled corn with antimicrobial activity against *L*. *monocytogenes*, *L*. *innocua*, and *E*. *faecalis*. *L*. *plantarum* strain LR/14 identified by Tiwari et al.^110^ was characterized to produce a bacteriocin inhibiting *L*. *monocytogenes*, *S*. *aureus*, and urogenic *E*. *coli*. Song et al.^111^ indicated that plantaricin ZJ5 (PZJ5) produced by *L*. *plantarum* ZJ5 with a molecular weight of 2572.9 Da had strong activity against *S*. *aureus*. The antimicrobial activity of nine bacteriocins (ET05, ET06, ET12, ET30, ET31, ET32, ET34, ET35, and ET88) of bacteriocin‐producing LAB isolated from vacuum‐packaged cold‐smoked salmon (CSS) was determined against *S*. *aureus*, *E*. *faecalis*, *L*. *monocytogenes*, and *E*. *faecium* in a study by Tome et al.^112^ In their study, bacteriocin ET06 belonging to the Class IIa bacteriocins inhibited 84.2% of 19 *Listeria* strains. The highest anti‐*Listeria* activity was recorded for ET05, ET30, and nisin derived from *L*. *lactis* subsp. *lactis* ATCC 11454.^112^ In another study in 2015, bacteriocins produced by *L*. *curvatus* MBSa2 (sakacin P) and *L*. *curvatus* MBSa3 (sakacin X) were shown to have inhibitory activity against *L*. *monocytogenes* strains. Bacteriocin produced by *L*. *curvatus* MBSa2 caused a 2 and 1.5 log reduction in the count of *L*. *monocytogenes* in the salami after 10 and 20 days, respectively, suggesting that application of these bacteriocins could be an additional measure to improve the safety of these ready‐to‐eat products with regards to *L*. *monocytogenes*.^113^ In a study by Mokoena (2017), bacteriocins ST22Ch, ST153Ch, and ST154Ch, produced by *L*. *sakei* strains (ST22Ch, ST153Ch, and ST154Ch) isolated from Salpicao, were shown to have inhibitory activity against *Listeria* spp., *Enterococcus* spp., *Klebsiella* spp., *E*. *coli*, *Pseudomonas* spp., *Staphylococcus* spp., and *Streptococcus* spp. Maximum activity of these bacteriocins was recorded during the early stationary phase. All sakacins belonged to the Class IIa bacteriocins and possessed strong antilisterial activity. Bacteriocins ST22Ch, ST153Ch, and ST154Ch were produced at high levels during all phases of (fermented) meat processing. The spectrum of antibacterial activity of these strains (ST22Ch, ST153Ch, and ST154Ch) indicates their potential application in a mixed starter culture for the fermentation of meat products.^5^ Sivakumar et al.^114^ showed that bacteriocin of *L*. *acidophilus* (isolated from milk) inhibited the growth of foodborne pathogens, such as *V*. *cholerae*, *S*. *typhi*, *Shigella* spp., *B*. *cereus*, *S*. *aureus*, *E*. *coli*, and *E*. *faecalis*. In a study by Yi et al. (2016), bacteriocin MN047A (BMA) was found to have antibacterial activity against multidrug‐resistant bacteria and broad‐spectrum inhibitory activity against both Gram‐positive and Gram‐negative bacteria, such as *E*. *coli* and *S*. *aureus*. This bacteriocin was produced by *L*. *crustorum* MN047, which was first isolated from koumiss.^115^ In another study by Jiang et al. (2017), a novel bacteriocin called pentocin JL‐1 was produced by *L*. *pentosus*; this bacteriocin showed a broad‐spectrum inhibitory activity against both Gram‐positive and Gram‐negative bacteria, especially multidrug‐resistant *S*. *aureus*. These results suggest that pentocin JL‐1 has the potential to be used as a biopreservative in the food industry.^116^ Zhu et al. (2000) isolated bacteriocin‐producing *L*. *gasseri* strain KT7 from infant feces. The bacteriocin gassericin KT7 showed inhibitory activity against some pathogenic bacteria including *Listeria*, *Clostridium*, and *Enterococcus*. This bacteriocin was produced constitutively during exponential growth phase.^117^ Another bacteriocin, produced by *L*. *viridescence*, was identified by Kp et al.^118^ for use in the preservation of various food products with strong antibacterial properties. For the first time, Gautam et al. (2015) reported bacteriocin‐producing potential of *L*. *spicheri* strains. Bacteriocin of *L*. *spicheri* G2 showed strong activity against pathogenic bacteria such as *S*. *aureus*, *L*. *monocytogenes*, *C*. *perfringens*, *S*. *mutans*, *L*. *mesenteroides*, and *B*. *cereus*. Maximum activity of this bacteriocin (2000 AU/ml) was recorded in MRS broth at 35°C and pH 4.0 after 34 h.^119^ *L*. *acidophilus* produces Class II acidocin CH5 with a restricted activity against Gram‐positive bacteria. Deraz et al.^120^ identified another bacteriocin designated as acidocin D20079, produced by *L*. *acidophilus* DSM 20079, with a low molecular weight (6.6 kDa), extreme pH‐ and heat‐stability, and narrow‐spectrum inhibitory activity. Table 1 summarizes the classification of bacteriocins with few examples.

**TABLE 1:** Bacteriocins of *Lactobacillus* and their main characteristics

Bacteriocin	Species and strain	Source	Mol. wt. (kDa)	Heat‐stability range	Sensitivity	PH Stability range	Production phase	Optimal production	Inhibitory‐activity spectrum	Reference
ET05, ET12, ET32, ET34,	*L. delbrueckii*	Cold‐smoked salmon	3.5	At 60°C for 60 min, and at 100°C for 20 min.	Proteolytic enzymes (pepsin A)	2–8	MRS broth with 0.4% DL‐threonine at 30°C	Heat: 37°C PH: 6.5	*L. monocytogenes*	112
ET06, ET30, ET31,	*L. curvatus*	Cold‐smoked salmon	2.8–4.5	At 60°C for 60 min, and at 100°C for 20 min.	Proteolytic enzymes (pepsin A)	2–8	MRS broth with 0.4% DL‐threonine at 30°C	Heat: 37°C PH: 6.5	*L. monocytogenes*	112
Bacteriocins MBSa2 and MBSa3	*L. curvatus*	Salami	2–4	At 25 and 37°C for 8 h, at 30°C for 6 h, and at 121°C for 15 min	Proteolytic enzymes: a‐chymotrypsin Streptomyces griseus protease type XIV Trypsin Pepsin Proteinase K	2–10	MRS agar at 37°C for 24 h	Heat: 37°C PH: 6–8	*L. monocytogenes*	113
Fermenticin HV6b	*L*. *fermentum* HV6b MTCC 10770	Human vaginal ecosystem	(−)	(−)	(−)	(−)	MRS supplemented with 0.1% w/v Tween 80, at pH 6.5 and 37°C	(−)	*B. fragilis* *B. ovatus* *B. vulgatus* *C. albicans* *C. sporogenes* *E. coli* *E. faecalis* *G. vaginalis* *K. pneumoniae* *L. mesenteroides* *L. monocytogenes M. flavus* *N. gonorrhoeae* *N. mucosa* *P. aeruginosa* *P. mirabilis Staphylococci Streptococci* *S. typhi* *V. cholerae*	121
Nisin	*L*. *lactis* subsp. *lactis* ATCC 11454	Milk and traditional Sardinian cheese		At 121°C for 15 min		2.5–9			*L. monocytogenes* *C. difficile* *S. pneumoniae* *E. faecalis* *S. bovis* *S. aureus*	122
ST22Ch,	*L. sakei*	Pork product	3.0	At 30–100°C for 2 h	Proteolytic enzymes: Proteinase K Pronase Trypsin Pepsin Papain	2–10	MRS agar at 30°C for 24 h		*Enterococcus* spp. *Listeria* spp. *E. coli* *Klebsiella* spp. *Pseudomonas* spp. *Staphylococcus* spp. *Streptococcus* spp.	123
*Bacteriocin MN047*	*L*. *crustorum* MN047	Koumiss from Xinjiang	1.77	Up to 121°C for 30 min	Protease	2–11	MRS agar at 37°C for 48 h		*S. aureus* *E. coli*	115
Bacteriocin GM3	*L*. *sakei* GM3	Goat milk	4.8	At 75°C for15 min, at 85°C for 15 min, at 85°C for 30 min, and at 100°C for 20 min	Pepsin Trypsin Papain Proteinase K	2–10	MRS medium at 37°C		*P*. *aeruginosa* MTCC 741 *S*. *aureus* MTCC 3160	124
Plantaricin	*L*. *plantarum* strain EC52	Poto, an ethnic maize fermented food	(−)	(−)	(−)	(−)	MRS Spain at 30°C		*L*. *monocytogenes E*. *coli* O157:H7	125
Plantaricin JLA−9	*L. plantarum* JLA−9	Suan‐Tsai	1.04	At 121°C for 20 min	α‐chymotrypsin Pepsin Alkaline protease Papain	2–7	MRS 92 medium at 37°C		*Bacillus spp*.	102
Gassericin KT7	*L*. *gasseri* KT7	Infant feces	5	At 100 °C for 30 min, and at 121°C for 30 min	Proteinase K Trypsin Chymotrypsin ^®^cin Papain	2–10	MRS broth at 35°*C* for 34 h	Heat: 30–70*°C* PH: 4–7	*C. perfringen* *C. botulinum* *S. aureus* *L. monocytogenes E. facecalis* *B. cereus*	117
Pentocin JL−1,	*L. pentosus*	Grey carpet shark	2.9	At 120°C for 15 min	Proteinase K Trypsin Pepsin Alkaline protease	5–7	MRS at 30 ∘C for 24 h	Heat: 30*°C* PH: 5–6	Multidrug‐resistant *S*. *aureus*	116

#### 11.1.2 Bacteriocin of *Enterococcus*

*Enterococcus* is the third main genus of lactic acid bacteria (LAB) after *Lactobacillus* and *Streptococcus*.^126^ They are ubiquitous bacteria of human and animal gastrointestinal tracts, which are also present in vegetables, meat, milk, and cheese.^127^ Their ability to colonize such diverse ecological niches is related to their tolerance in harsh environmental conditions.^128^ Despite concerns about their existence as important opportunistic pathogens causing a wide variety of infections, they could exhibit interesting functions. Purification and genetic characterization of many enterococci‐derived peptide bacteriocins have been reported, most of which have been obtained from *E*. *faecalis* and *E*. *faecium* (Table 2). Since some of these bacteriocins could not be grouped with typical LAB‐derived bacteriocins according to traditional classification, enterocins classification into four new classes has been proposed.^129^ Cytolysin produced by *E*. *faecalis* is a two‐linear peptide lantibiotic with cytolytic (hemolytic) activity, and its encoding genes are found in both hospital and food isolates. It differs structurally from other linear lantibiotics including nisins A and Z.^130^, ^131^ Class II enterocins are two‐peptide bacteriocins of the pediocin family.^132^ The most common antimicrobial peptides are pediocin‐like enterocins, also known as Class II‐1 bacteriocins, which are classified into two subgroups based on sequence similarity.^133^ All of them have a conserved YGNGVXC “pediocin box” motif and a β‐sheet domain supported by a conserved disulfide bridge at the N‐terminus.^134^ Enterocin A, mundticins, and enterocin CRL5 are among the first subgroup.^129^ The second subgroup is represented by bacteriocin 31, bacteriocin T8, bacteriocin RC714, enterocin SEK4, enterocin P, and enterocin M.^129^ Class II‐2 bacteriocins lack the YGNGVXC motif and are not synthesized with a leader peptide; however, they must have complementary actions of both peptides to be completely active.^13^ A main subgroup includes enterocin Q, enterocin C, two‐peptide bacteriocin L50 (A, B), enterocin RJ‐11, and enterocin EJ97.8. Other linear non‐pediocin‐like enterocins with a leader peptide are classified as Class II‐3. The presence of a second peptide may reinforce their antimicrobial activity. Class III enterocins are cyclic peptides including enterocin AS‐48 produced by *E*. *faecalis* S‐48. Finally; Class IV includes a large and heat‐labile bacteriocin, named enterolysin A produced by *E*. *faecalis*. Enterocins have a wide range of antimicrobial activity, inhibiting not only the growth of closely associated bacteria but also Gram‐positive pathogens such as *L*. *monocytogenes*, *S*. *aureus*, and *V*. *cholerae* in food. This particularity has been observed for example in enterocin LD3, enterocin CV7, enterocin B, enterocin Gr17, enterocins A and B, and enterocin dP.^129^ Besides, these bacteriocins show broad‐spectrum antimicrobial activity against Gram‐negative bacteria such as *E*. *coli*, *Salmonella* sp., *Psudomonas* spp., and *Vibrio* sp.^135^, ^136^, ^137^, ^138^ Also, they are considered as one of the weapons against multidrug‐resistant pathogens and prevent biofilm formation. The main multidrug‐resistant organisms include ESBL‐producing *Enterobacteriaceae*, MRSA, and VRE.^60^, ^139^ Despite their potent activity against foodborne pathogens, enterococcal bacteriocins have not yet been approved for use in natural food preservation due to safety concerns.^126^ Recently, *E*. *faecium* SF68^®^ (NCIMB 10415, Cerbios‐Pharma SA, Barbengo, Switzerland) and *E*. *faecalis* Symbioflor 1 (SymbioPharm, Herborn, Germany) are commercially available on the market as two probiotic strains.^129^, ^140^

**TABLE 2:** Bacteriocins of *Enterococcus* and their main characteristics

Bacteriocin	Production strain	Source	Molecular mass	Heat‐stability range	PH‐Stability range	Stability	Sensitivity	Production parameter	Inhibitory‐activity spectrum	Reference
Enterocin LD3	*E*. *hirae* LD3	Dosa	4.1	At 121°C for 30 min	2–6	Amylase lipase	Papain Proteinase K Pepsin Trypsin	MRS	*S. aureus* *P. fluorescens* *P. aeruginosa* *S. typhi* *S. flexneri* *L. monocytogenes* *E*. *coli* O157:H7 *E*. *coli* (urogenic, a clinical isolate) *Vibrio* sp.	(135, 136)
Unnamed	*E.mundtii* QAUEM2808	Fermented milk product Dahi	(−)	(−)	(−)	(−)	(−)	TSB	*E*. *coli* ATCC 10536 *S*. *aureus* ATCC 6538 *P. aeruginosa* ATCC 9027 *L. monocytogenes* ATCC 13932	138
Enterocin B	*E*.*faecium* YT52	Boza	5.5	At 100°C for 20 min	5.0–10.0	Lipase Lysozyme Catalase	Proteinase K Trypsin a‐chymotrypsin Pepsin	GM17 (10 h) logarithmic growth phase (7 h)	*L. monocytogenes B. cereus*	141
Nisin	*E. hirae* DF105Mi	Goat milk	(−)	At 121°C for 20 min	2.0–12.0	Detergents α‐amylase Catalase	Proteinase K Pronase Trypsin Pepsin Papain	MRS, exponential phase	*L. monocytogenes*	142
Faerocin MK	*E*.*faecium* M3K31	Vigna mungo	N.D	At 100 °C for 30 min	2–12	NaCL	Proteinase K Papain	MRS (18 h)	*L. monocytogenes* *S. aureus* *E. coli* *E. herbicola* *B. subtilis* *B. cereus* *P. aeruginosa*	143
TJUQ1	*E.faecium*	Pickled Chinese	(−)	4–45°C	6–9	(−)	(−)	MRS Overnight under static condition	*L*. *monocytogenes* CMCC 1595	144
KT11”	*E*. *faecalis* KT11	Cheese	~3.5	At 121°C for 30 min	2–11	Surfactants Solvents	Proteinase K α‐chymotrypsin Protease Trypsin	MRS (24 h)	Vancomycin‐ and/or methicillin‐resistant bacteria *L. monocytogenes* *S*. *aureus* ATCC25923 *B. subtilis* *S. aeruginosa,* *K. pneumoniae,* *S. marcescens* *E. aerogenes*	60
Enterocin CV7	*E*. *faecalis* CV7	Chicken	4.5	At 121°C for 10 min	2–12	Solvents Detergents	Pepsin Proteinase K Trypsin Chymotrypsin Catalase Lipase	MRS (24 h)	Gram‐positive bacteria: *L. monocytogenes* *S. aureus* Gram‐negative bacteria: *Salmonella* sp. *S. thypi* *S. enterica* *E. coli* *V. fischeri*	137
EF478	*E. faecalis*	Water	45	2–8	At 60°C for 1 h, at 4°C for 6 months, at 25°C for 2 months, and at −20°C for one year	Amylase Lipase	Proteinase K Trypsin α‐chymotrypsin	MRS (16 h) PH: 5–6	*E*. *faecalis* ATCC 51299 *E*. *faecium* ATCC 35667	145
Enterocin dP	*E. lactis* Q1	Shrimp	(−)	4.0–9.0	At 100°C for 15 min	(−)	Proteinase K Trypsin	MRS Aerobic conditions	*L. monocytogenes* *P. aeruginosa* *L. garvieae* Fungi (*Aspergillus niger* and *Fusarium equiseti*)	146
B3A‐B3B	*E*. *faecalis* B3A‐B3B	Feces	5.2	2–10	(−)	CFS	Proteinase K Trypsin Papain Lipase Catalase Lysozyme	MRS (24 h)	*L. monocytogenes* *S. aureus* Methicillin‐resistant *S*. *aureus* (MRSA) *C*. *perfringens*	139
152A and L50B	*E*. *durans* 152	Floor	5	2–8	At 121 ℃ for 15 min	(−)	Proteinase K	TSB (24 h)	*L. monocytogenes*	147
DU10	*E.faecalis* DU10	Duck intestine	6.3	2–12	At 121°C for 10 min	Detergents Solvents	Proteinase K Pepsin Trypsin α‐chymotrypsin	MRS	Gram‐positive bacteria: *L. monocytogenes* *S. aureus* Gram‐negative bacteria: *S. typhi* *S. enterica Salmonella sp. E.coli*	148
Enterocin A, B	*E*.*faecium* GGN7	Cow milk	4.8 and 5.4	2–8	At 121°C for 10 min	(−)	Proteinase K Trypsin	MRS (16 h)	*L*. *ivanovii* BUG 496 *P*. *aeruginosa* 27853	149
F4‐9	*E.faecalis* F4‐9	Red fish	5.5	2 – 6	At 100°C for 15 min	(−)	Trypsin Chymotrypsin Proteinase K	MRS (16 h)	A Gram‐negative strain *Enterococcus* *B. coagulans* *E*. *coli* JM109	150
CRL 1826	*E. gallinarm* CRL 1826	Captive bullfrog	(−)	2–9	At 80°C for 30	Solvents Lipase α‐amylase	a‐chymotrypsin Trypsin Pepsin	MRS Microaerophilic conditions	*Citrobacter freundii P. aeruginosa* (bullfrog pathogens) *L. monocytogenes*	151
LD3	*E*. *hirae* strain LD3	Dosa batter	Between 3.48 and 7.68	2 – 6	At 121°C for 15 min	Solvents Detergents Catalase Amylase Lipase	Pepsin Trypsin Proteinase K Papain	MRS (18 h)	*S. aureus* *P. fluorescens* *P. aeruginosa,* *S. typhi* *S. flexneri* *L*. *monocytogenes E*. *coli* O157:H7 *Vibrio* sp.	152
SH01	*E.faecium* SH01	Mukeunji	3	2–12	At 121°C for 15 min	α‐Amylase Catalase Detergents	α‐chymotrypsin Pronase E Proteinase K Trypsin	MRS Early stationary (24 h)	*L. monocytogenes* KCTC 3569 *L*. *curvatus* KFRI 166 *L*. *mesenteroides* ATCC 10830 (agar‐well‐diffusion assay)	153
FL31	*E.faecium*	(−)	3.5	2–9	At 100°C for 90 min	Surfactants Solvents	Trypsin Pronase E Proteinase K	MRS Early stationary	*L*. *monocytogenes* ATCC19117	154

#### 11.1.3 Bacteriocins of *Streptococcus*

The genus *Streptococcus* embraces a broad range of Gram‐positive bacteria, some of which are pathogenic species, while others are important for use in dairy products fermentation. *S*. *thermophilus* is the only streptococcal species that along with *Lactobacillus* spp. is used as an industrial dairy starter culture, especially for yogurt manufacturing.^155^ It could improve the quality of fermented dairy products through various probiotic effects and the production of bacteriocins, flavor substances, and extracellular polysaccharides.^156^ Renye et al.^157^ demonstrated that *S*. *thermophilus* strain ST109 produced an antimicrobial peptide against lactobacilli, enterococci, and human pathogen *S*. *pyogenes*. Various studies have reported the ability of *Streptococcus* spp. against human pathogens and common foodborne bacterial pathogens^158^, ^159^, ^160^ (Table 3). In another study by O'Connor et al.,^161^ *S*. *hyointestinalis* DPC6484 isolated from the porcine intestine was shown to produce a bacteriocin designated as nisin H with a molecular mass of 3,453 Da, exhibiting an antimicrobial activity against a wide range of Gram‐positive genera. The use of nisin variants as natural food preservatives has been reported in over 50 countries worldwide without inducing microbial resistance.^162^

**TABLE 3:** Bacteriocins of *Streptococcus* and their main characteristics

Bacteriocin	Production strain	Source	Molecular mass	Stability	Sensitivity	Production parameter	Inhibitory spectrum	Reference
Nisin H	*S. hyointestinalis* DPC6484	Porcine intestine	3.4	(−)	(−)	TSB at 37°C	*E*. *coli* DPC6912 *B*. *cereus* 9139 *L*. *delbrueckii* subsp. *bulgaricus* LMG6901 *L*. *lactis* subsp. *cremoris* HP *E*. *faecalis* 6307 *S*. *agalactiae* ATCC 13813 *S*. *agalactiae* DPC5338 *S*. *bovis* DPC6491 *S*. *gallolyticus* DPC6501 *L*. *innocua* DPC3572 *L*. *monocytogenes* 1042 *S*. *aureus* ATCC 25923 *S*. *aureus* DPC5245 *S*. *hyointestinalis* DPC6484 *S*. *uberis* strain 42	161
Unknown	*Streptococcus* species (G3, G4)	Green gram	(−)	PH: 5	(−)	MRS broth at 37°C for 48 h	*P. aeruginosa* *S. aureus* *E. coli* *Klebsiella*	160
Thermophilin 109	*S. thermophilus* ST109	Raw milk	5–6	At 121°C for 17 min.	*B*. *subtilis* protease Proteinase K Trypsin a‐amylase	TYL broth for 18 h	In TYL broth: Other *S*. *thermophilus* strains *L*. *delbreuckii* ssp. *bulgaricus* *L. acidophilus* *S. pyogenes* In M17G broth: *E. faecalis* *E. faecium*	157
Suicin 65	*S*. *suis* 65	Pigs	3.3 (SDS‐PAGE)	Enzyme: trypsin, chymotrypsin, proteinase K/30 min PH: 2 and 11 Heat: 121°C for 15 min	(−)	Todd Hewitt agar (THA) plates supplemented with 0.01% Tween 80	*S*. *suis* strains from different countries	158
Suicin 3908	*S*. *suis* serotype 2	Pigs	3.3 (SDS‐PAGE)	Enzymes: trypsin, chymotrypsin, proteinase PH: 2 and 11 Heat: 121°C for 15 min	(−)	2 L of a culture medium containing 2% proteose‐peptone, 1% yeast extract, 0.25% glucose, 0.25% NaCl, 0.3% K_2_HPO_4_, 0.2% KH_2_PO_4,_ 0.01% MgSO_4_ · 7 H_2_O, and 0.002% MnSO_4_ · 7 H_2_O (pH 7.0)	*S. hyicus*	159

#### 11.1.4 Bacteriocins of *Pediococcus*

*Pediococcus* is a type of lactic acid bacteria, which plays a major function in the preservation of fermented food products such as vegetables, meat, sausage products, and cheddar cheese.^163^ Bacteriocins of *Pediococcus* species are designated as pediocins and produced by three species that play an important role in food biopreservation, including *P*. *acidilactici*, *P*. *cerevisiae*, and *P*. *pentosaceus*. They generally belong to the Class IIa bacteriocin family with a small molecular weight (<5 kDa); the identified N‐terminal region of all pediocins includes a pattern known as "pediocin box." They show a wide range of antimicrobial activity against other bacteria related to food spoiling and health jeopardies of food source. The biological and chemical characteristics of pediocins are shown in Table 4. Various species are stable in a broad span of pH and temperature, which makes them suitable for biopreservation of various food products.^164^ Production of bacteriocin usually occurs during the stationary growth phase in MRS broth. Several studies have reported that both *P*. *acidilactici* and *P*. *pentosaceus* are associated with food fermentation and produce various pediocins which inhibit several food spoilage and human pathogenic bacteria.^32^ Pediocins are Class IIa bacteriocins produced by *Pediococcus* spp., which are commercially accessible under the name Alta 2341TM or Microgard TM. These bacteriocins are more effective than nisin against certain foodborne pathogens, such as *L*. *monocytogenes* and *S*. *aureus*, and Gram‐negative organisms such as *Pseudomonas* and *E*. *coli*. The potential application of pediocins in dairy products is further enhanced by considering their stability in aqueous solutions over a wide pH range and in heating or freezing systems. Despite this high possibility, a few studies have examined the addition of pediocins to milk or dairy products. Pediocin PA1 was found to reduce *L*. *monocytogenes* numbers in cottage cheese, cream, and cheese sauce by Garsa et al.^165^

In a study, pediocin PA‐1 was derived from *P*. *acidilactici* PAC1 and shown to lyse a strain of *L*. *monocytogenes*.^166^ Choi and Beuchat^167^ isolated a bacteriocin from *P*. *acidilactici*, which had a deadly result on *L*. *monocytogenes*. Jamuna and Jeevaratnam^168^ extracted a bacteriocin from *P*. *cerevisae* P18, which was composed of a combination of proteinaceous materials and was active against *L*. *monocytogenes*. Pediocin A was obtained in a study from *P*. *pentosaceus* with a molecular mass of 80 kDa, which had inhibitory activity against *Lactobacillus*, *Leuconostoc*, *Pediococcus*, *Staphylococcus*, *Enterococcus*, *Listeria*, and *Clostridium* species.^169^ A new bacteriocin was obtained in a study from *P*. *pentosaceus* ACCEL with a molecular mass of 17.5 kDa and stability at pH 2.0–6.0; only Gram‐positive bacteria were inhibited by this bacteriocin.^165^ A new proteinaceous compound with a molecular mass of 23 kDa has been derived from *P*. *pentosaceus* for the first time, which is active against *S*. *dysenteriae*; this bacteriocin could be supplemented into any food to make it safe against *S*. *dysenteriae* as a foodborne pathogen causing alarm in many developing countries.^170^ A pediocin‐producing strain of *P*. *acidilactici* able to survive in the GI tract has been recently isolated and found to be an effectual inhibitor of various Gram‐positive bacterial pathogens, such as *Enterococcus* spp. and *L*. *monocytogenes*. It demos inhibitory activity against gastric adhesion of opportunistic pathogens, such as *Klebsiella*, *Pseudomonas*, and *Shigella* species.^171^ Speelmans et al. (2006) isolated *P*. *pentosaceus* 05–10 from a traditionally fermented Sichuan pickle, which could produce a bacteriocin designated as pediocin 05–10 active against *Listeria*, *Lactobacillus*, *Streptococcus*, *Enterococcus*, *Pediococcus*, *and Leuconostoc*. The broad anti‐Listeria activity exhibited by pediocin 05–10 is the characteristic of Class IIa bacteriocins. Pediocin 05–10 was sensitive to proteolytic enzymes but stable at pH 2–10 and resistant to heat (15 min at 121°C).^171^

**TABLE 4:** Bacteriocins of *Pediococcus* and their main characteristics

Bacteriocin	Species	Source	Mol. wt. (kDa)	Heat stability range	Sensitivity	PH Stability range	Production phase	Optimal Production	Inhibitory activity spectrum	Reference
VJ13B	*P. pentosaceus VJ13*	Idli batter	4.0	At 100°C for 1 h	Protease	2–8	MRS broth (24 h)	Heat: 37°C PH: 5.0–6.0	*M. smegmatis* *K. pneumonia L.monocytogenes* *C. perfringens* *S. epidermidis*	172
NKSM1	*P. pentosaceus* NKSM1	Appam batter	9–14	At 110°C for 2 h	Trypsin Papain	4–7.5	MRS broth (48 h)	Heat: 30°C PH: 6.0	*L*. *monocytogenes* MTCC 657 *A*. *baumannii* MTCC 1425	173
A11 and C12	*P*. *acidilactici* A11 and C12	Breast milk isolate (ASI)	(−)	(−)	(−)	(−)	MRS broth (24 h)	Heat: 37°C	MRSA	174
*BLIS*	*P*. *pentosaceus* ATCC 43200	Shake flasks to bioreactor	75	At 121°C for 15 min	(−)	3.8–6.5	MRS broth (16 h)	Heat: 37°C PH: 6.0 ± 0.2 Addition of 1.0 N HCl	*L. innocua* *L. seeligeri*	41
Pediocin L50	*P*. *acidilatici* FS6	Human breast milk	2.7–17	(−)	(−)	2.5	MRS broth (24 h)	Heat: 37°C PH: 2.5	*E*. *coli* 0157:H7 *L*. *monocytogenes* ATCC 15313 *B*. *cereus* ATCC 14576 *S aureus* ATCC 19095 *H. pylori*	175
PE‐ZYB1	*P*. *pentosaceus* zy‐B	Intestine of *Mimachlamys nobilis*	2.02	At 121°C for 20 min	Trypsin	2–10	MRS broth (48 h)	Heat: 37°C PH: 2–7	*L.monocytogenes*	49
(−)	*P. pentosaceus*	Brazilian artisanal cheese	(−)	At 121°C for 15 min	Proteinase K Trypsin Pepsin α‐Chymotrypsin Protease type XIV	2–10	MRS broth (24 h)	Heat: 37°C PH: 6.5	*L. monocytogenes* 211 *L*. *monocytogenes* 422	51
(−)	*Pediococcus acidilactici* K10	Kimchi	(−)	(−)	(−)	2.5–4	MRS broth (24 h)	Heat: 37°C PH: 3.0	*S. typhimurium* *E*. *coli* O157:H7	176
Pediocin	*P*. *acidilactici* CFR K7	Pickles and dry‐cured meats	5	At 121°C for 20 min	Pepsin Trypsin Proteinase K	2–11	MRS broth (48 h)	Heat: 30°C PH: 6.0	*S. aureus* *E. coli*	177
	*P. acidilactici*	Gappal			Protease Pepsin Trypsin		MRS broth (12 h)	Heat: 30°C PH: 6.0	*E*. *faecalis* ATCC 19433 *M*. *luteus* ATCC 49732 *S*. *aureus* ATCC 2523 *L. monocytogenes* *B. megaterium* *B. sphaericus* *B. cereus*	

#### 11.1.5 Bacteriocins of *Carnobacterium*

*Carnobacterium* spp. isolated from poultry, fish, and vacuum‐packaged meat were initially accepted as non‐aciduric *lactobacilli* by Parada et al.^178^ The carnobacterial bacteriocins belong to the Class I and II bacteriocins.^179^ Many studies have reported bacteriocins associated with this genus, which inhibit the growth of spoilage microorganisms in foodstuffs.^180^, ^181^ MMF‐32 and KOPRI 25789 are produced by *C*. *maltaromaticum* KOPRI 25789 and *C*. *maltaromaticum* MMF‐32 and show antimicrobial activity against *L*. *monocytogenes* and Gram‐negative bacteria in smoked salemon. High bacteriocin production occurs at 30°C and optimal pH 5–7. Additional experiments have indicated that producer isolates effectively inhibit foodborne pathogens *in vitro*, presumably due to the effects of bacteriocins.^182^, ^183^ Their resistance and/or sensitivity to proteases, general pH, and heat confirm the presence of bacteriocins and their potential usage as biopreservatives in food products (Table 5).

**TABLE 5:** Bacteriocins of *Carnobacter* and their main characteristics

Bacteriocin	Species and strain	Source	Mol. wt. (kDa)	Heat stability range	Sensitivity	PH Stability range	Production phase	Optimal production	Inhibitory activity spectrum	Reference
MMF−32 and KOPRI 25789	*C*. *maltaromaticum* MMF−32 *C*. *maltaromaticum* KOPRI 25789	Smoked salmon	—	At 56°C for 30 min and at 100°C for 10 min	Lysozyme Proteinase K Trypsin α‐Chymotrypsin	3.5–6.5	MRS broth (48 h)	Heat: 30°C, PH: 6.5±0.2	*Shewanella baltica* OS185 *L*. *monocytogenes* ATCC 19114 *Aeromonas* HB−6 *A*. *hydrophila* HX201006‐3 *S. baltica* *S*. *baltica* OS678 *Serratia* I−113–31 *A*. *salmonicida* subsp. *achromogenes*	184
‐	*Chromobacterium aquaticum*	Zebrafish	—	At 100°C for 20 min	Protease Xylanase	2–11	TSB agar (30 h)	Heat: 30°C PH: 5–7	*A. hydrophila* *S. iniae*	182
Piscicolin 126, carnobacteriocin BM1, and carnocyclin A	*C. maltaromaticum*	Lean beef	—	At 100°C for 20 min		2–11	LB broth (48 h)	Heat: 30°C PH: 5.4	*E*. *coli* AW1.7 *S. enterica Typhimurium*	183

#### 11.1.6 Bacteriocins of *Leuconostoc*

*Leuconostoc* spp. are lactic acid bacteria that are most commonly associated with fermented food products, such as fermented sausages, fermented vegetables, cereal products, and a wide variety of fermented milk products.^185^, ^186^ They are environmental organisms that are generally found in fresh plants, raw milk, or chilled food products.^185^ *Leuconostoc* spp. have the ability to produce aroma compounds (diacetyl and acetoin) in dairy products by metabolizing citrate.^187^ Antimicrobial activity of these organisms has long been recognized.^188^ Several studies have identified bacteriocin‐producing strains of leuconostocs (Table 6).^189^, ^190^, ^191^ Most *Leuconostoc* bacteriocins are classified as Class II, which are small (less than 10) and heat stable. They are non‐lanthionine‐containing bacteriocins without post‐translational modification.^16^ Several strains of *Leuconostoc* spp. could produce bacteriocin (Table 6). According to Table 6, *L*. *mesenteroides* is the most common bacteriocin‐producing species. They mainly prevent the growth of Gram‐negative and Gram‐positive bacteria, including *E*. *coli*, *Salmonella*, *Listeria* spp, and *S*. *aureus*, which are foodborne pathogens. Therefore, this species is considered as a good candidate for the use in fermented food products in the food industry.

**TABLE 6:** Bacteriocins of *Leuconostoc* and their main characteristics

Bacteriocin	Production strain	Source	Mol. wt. (kDa)	Heat‐stability range	Stability	pH Stability range	Sensitivity	Production parameter	Inhibitory activity spectrum	Reference
BacCHBY46	*L*. *mesenteroides* CHBY 46	Algerian dromedary milk	3.5 (Tricine‐SDS‐PAGE)	80–100°C	(−)	PH: 2–6	Protease α‐chymotrypsine Trypsine	MRS broth	*S*. *aureus* ATCC 29213 *P. aeruginosa* *L*. *innocua* ATCC 33090 *E*. *coli* ATCC 25922 *S*. *aureus* ATCC 29213 *K*. *oxytoca* ATCC 13182 *E*. *cloacae* ATCC 13047 *E*. *faecalis* ATCC 10541 *B*. *cereus* ATCC 10876 *P*. *aeruginosa* ATCC 27653	192
Unknown	*Leuconostoc* spp.	Stored meat	(−)	(−)	(−)	(−)	(−)	MRS broth	*E. coli* *S. typhimurium* *S. aureus* *L. monocytogenes*	193
*L. lactis* SD501	*L*. *lactis* SD501	Kimchi	7	At 121 ℃ for 15 min	Catalase Amylase Lipase Pepsin	1–10	Proteinase K α‐chymotrypsin	Early stationary phase in MRS broth at 37°C	*L. monocytogenes* *E. faecalis*	190
Unknown	*L. pseudomesenteroides*	Tibetan yaks	(−)	(−)	(−)	(−)	(−)	MRS broth for 24	*E. coli* *S. aureus* *S. enteritidis* *S. aureus*	194
Unknown	*L*. *lactis* BT17	Boza	(−)	(−)	(−)	(−)	(−)	MRS broth	*E*. *coli* ATCC 25922 *Salmonella* sp. *K. pneumoniae*	195
Leucocin K7	*L. mesenteroides*	Pickles	(−)	0–100°C	(−)	2–10	Pepsin Protease K Papain	MRS at 30°C for 24 h	*L*. *monocytogenes* strains CMCC54005 *S*. *aureus* ATCC 29213 *P*. *aeruginosa* ATCC 27653	196
BLIS 213M0	*L*. *mesenteroides* subsp. *dextranicum*	Mongolian fermented mare milk and airag	2.6–3. (SDS‐PAGE)	25°C for 1 week 121°C for 15 min	(−)	2 −11 4.5	Pepsin Trypsin α‐Chymotrypsin Proteinase K	MRS broth at 25°C exponential growth phase of the producer cells	*Listeri*a spp. *E. faecalis* *L. mesenteroides* *P. pentosaceus* *S. thermophilus*	189
Unknown	*L. mesentroides*	Domestic goat	(−)	(−)	(−)	4.2–9.2	(−)	MRS broth at 35°C for 24 h PH: 6.2	*S*.*aureus* ATCC25923 *E*.*coli* ATCC25422 *P*.*aeruginosa* ATCC27583 *K*.*pneumonia* ATCC700603	191
Mesentericin W3	*L. mesenteroides* subsp. *cremoris*	Wine	3.9 (MALDI‐TOF MS)	(−)	Acetonitril 2‐propanol Methanol Acetone Chloroform Ethanol	2 and 7	Proteinase K Trypsin	MRS broth pH 6.5 at 30°C	*Lactobacillus* *Leuconostoc* *Carnobacterium* *Listeria* *Enterococcus*	197
Unknown	*L. mesenteroides* strain P45	Pulque, a Mexican traditional alcoholic beverage	(−)	(−)	Lysozyme Bile salts	3.5	(−)	MRS or APT (DIFCO) broth at 30°C	*L*. *monocytogenes* Enteropathogenic *E*. *coli* *S*. *enterica* serovar *Typhi* *S*. *enterica* serovar *Typhimurium*	198
Unknown	*L. mesenteroides* Com−54	Kimchi	(−)	(−)	(−)	(−)	Trypsin Lysozyme	MRS broth	*Salmonella*	199
Homology with mesentericin Y105 (peak 1) and mesentericin B105	*L*. *mesenteroides* subsp. *mesenteroides* SJRP55 strain	Water Buffalo Mozzarella cheese	3.9 and 3.4 (Mass Spectrometry (MS))	At 100°C for 2 h 121°C for 20 min	Enzymes: α‐amylase Lipase Catalase Solvents: SDS Tween 20 Tween 80 Triton X−100 Na‐EDTA NaCl (1%) 1 M urea	2–4	Pepsin Protease Trypsin α‐chymotrypsin	MRS broth at 25°C Late log phase until the stationary phase	*Listeria* spp.	200
Unknown	*L*. *mesenteroides* subsp. *mesenteroid*es *L*. *mesenteroides* subsp. *Dextranicum*	Traditional date product "Btana"	(−)	At 55°C for 15 min	(−)	4.8–6.5	(−)	MRS broth	*P*. *aeruginosa* ATCC 27853 *E*. *faecalis* ATCC 29212 *E*. *coli* ATCC 25922 *S*. *aureus* ATCC 25923	201

#### 11.1.7 Bacteriocin of *Bifidobacterium*

Intestinal microbial balance could be improved by suppressing the growth of different potentially pathogenic microorganisms. The production of organic acids and the secretion of antimicrobial compounds such as bacteriocins may be induced by antimicrobial activity of bifidobacteria.^202^ Purified bifidocin A shows a wide span of antimicrobial activity against a great deal of food spoiling pathogenic bacteria such as *E*. *coli*, *L*. *monocytogenes*, *S*. *aureus*, and some yeasts^53^ (Table 7). Bifidocin A is produced in the exponential growth phase with the maximal production in the midi‐stationary phase. This suggests that bifidocin A production depends on the cell number, and bifidocin A is a secondary metabolite.^203^ The reduction in the activity of bifidocin A at the end of the stationary phase may be because of the activity of endogenous extracellular proteases, which peak pending this growth phase.^204^ A sensitive strain (*E*. *coli* 1.90) was tested in a study to determine whether bifidocin A has a bactericidal or bacteriostatic mode of action. The outcome displayed that the inhibitory activity of bifidocin A against *E*. *coli* 1.90 was bactericidal with cell lysis, which is consistent with that of bifidocin B.^205^ Antimicrobial activity of substances made by several *Bifidobacterium* strains (*B*. *bifidum* and *B*. *lactis* Bb‐12) is curbed to Gram‐negative pathogenic bacteria.^206^ Yildirim et al. (1999) showed that their application could be beneficial in the food commercial enterprise in modelling HACCP plans to effectively delete Gram‐negative pathogenic bacteria in meat products, especially *E*. *coli* 0157:H7 xx. Bifidocin B was made in a study in the exponential growth phase and reached a peak activity of 3,200 AU/mL at the primary stationary phase. Bifidocin B had a molecular mass of 3.3 kDa as analyzed by tricine‐sodium dodecyl sulfate‐polyacrylamide gel electrophoresis.^207^, ^208^

**TABLE 7:** Bacteriocins of *Bifidobacterium* and their main characteristics

Bacteriocin	Species and strain	Source	Mol. wt. (kDa)	Heat stability range	Sensitivity	PH Stability range	Production phase	Optimal production	Inhibitory activity spectrum	Reference
BB04 bifidocin A	*B.animals*	Healthy centenarians feces	1.9	At 100°C for 30 min	Trypsin Pepsin Proteinase K Acid proteinase Neutral proteinase	2–11	MRS broth^32^	Heat: 37°C PH: 8.0	*E. coli* *L. monocytogenes* *S. aureus*	53
Bifidin or bifilact Bb−12	*Bifidobacterium* *bifidum* and *B*. *lactis* Bb−12	Cow meat (round cuts) and fat	—	At 121°C for 15 min	—	4–10	MRS broth^16^	Heat:37°C PH: 5–7	*A. hydrophilia* *A. caviae* *A. sobria* *P. flouresent* *P. aeruginosa* *P. fragi* *E*. *coli* 0157:H7.	206
Bifidin I	*B*.*animalis* subsp*Lactis*	Cheese Whey	—	—	Proteolytic enzymes	6	MRS broth (48 h)	Heat:37°C PH: 6.0	*L*. *monocytogenes* ATCC *13932* *E*. *coli* ATCC 25922 *L*. *sakei* ATCC 15521	209
Bifidoadocin	*B. adolescentis*	Saliva of 18 volunteers postmenopausal women	14.6	At 121°C for 15 min	Pepsin Lipase α‐amylase Papain Chymotrypsin	2–10	MRS broth (24 h)	Heat:37°C PH: 6.0	*E*. *coli American* Type Culture Collection (ATCC) *S. aureus ATCC*	210
Bifidin I	*B*. *infantis* BCRC 14602	—	3	At 121°C for 15 min	—	4–10	MRS broth (18 h)	Heat:37°C PH: 5–7	LAB strains *Staphylococcus* *Bacillus* *Streptococcus* *Salmonella* *Shigella* *E. coli*	203

#### 11.1.8 Bacteriocins of *Lactococcus*

Nisin is categorized as a Class‐Ia bacteriocin or lantibiotic. To date, eight types of nisin have been observed and characterized. Nisins A, Z, F, and Q are produced by *L*. *lactis*, and nisins U, U2, P, and H are made by some *Streptococcus* strains.^211^, ^212^ Nisins effectively belong to the Class I bacteriocins that contain lanthionine and have a double mode of action: (i) they attach to lipid II which is the chief transporter of peptidoglycan subunits from the cytoplasm to the cell wall, inhibit precise cell wall synthesis, and induce cell death, and (ii) they take lipid II as a docking molecule to start the process of membrane insertion and pore formation, leading to speedy cell death.^212^ Nisin shows antimicrobial activity against some Gram‐positive bacteria, including LAB, pathogens such as *Listeria* and *Staphylococcus*, and spore‐forming bacteria such as *Bacillus* and *Clostridium* (Table 8). Nisin has been used extensively in cheese and pasteurized cheese spreads as an alternative to nitrate in order to hinder the outgrowth of clostridia spores.^211^ Some research has been undertaken to show the efficacy of nisin (or other bacteriocins) against *L*. *monocytogenes* in fresh products. Some studies have focused on combination treatments, for example, nisin in combination with acids and EDTA.^213^ The combined effect of nisin and bovicin HC5 against *L*. *monocytogenes* and *S*. *aureus* in fresh cheese was investigated by Pimentel‐Filho et al. (2014). They reported a significant diminution in *L*. *monocytogenes* count to an undiscernible level after 9 days of storage at 4°C; however, the combined effect of bacteriocins did not stop the growth of *S*. *aureus*.^214^ Józefiak et al.^215^ made use of a nisin‐supplemented bird diet to feed broiler chickens and found a decrease in the amount of *Bacteroides* and Enterobacteriaceae in ileal digesta of chickens fed by nisin‐supplemented diet. Nisin A and lacticin Q produced by *L*. *lactis* are more effective in reducing planktonic cells than methicillin‐resistant *S*. *aureus* biofilm cells.^216^ In addition to LAB bacteriocins, a non‐LAB bacteriocin, named sonorensin, was purified by Chopra et al.^217^ from *B*. *sonorensis* and displayed a significant anti‐biofilm activity against *S*. *aureus*. To date, only nisin (Nisaplin, Danisco) and pediocin PA1 (MicrogardTM, ALTA 2431, Quest) have been commercialized as food compounds.^218^ However, some other LAB bacteriocins propose promising outlook for use as biopreservatives, such as enterocin AS‐48^219^ or lacticin 3147^220^; no other bacteriocin has been proposed for industrial application. Lacticins are made by specific strains of *L*. *lactis* and include lacticin 3147 and lacticin 481. Lacticin 3147 powder was analyzed by Morgan et al.^221^ and found to be effective in controlling *Listeria* and *Bacillus* in infant milk formulation, natural yoghurt, and cottage cheese. Lacticin 481 is a single‐peptide lantibiotic with a moderate spectrum of inhibitory activity, which is primarily active against other LAB, *C*. *tyrobutyricum*, and *L*. *monocytogenes*. The utilization of semi‐purified lacticin 481 in fresh cheese stored at refrigeration temperatures was investigated by Ribeiro et al. (2016), leading to a 3‐log reduction in *L*. *monocytogenes* amount during 3–7 days. However, the use of lacticins in food products is unlikely to ensure full deletion of pathogens such as *L*. *monocytogenes*.^222^ Lactococcin BZ is made by some strains of *L*. *lactis* spp. (lactis BZ) and shows broad antibacterial activity against Gram‐positive and Gram‐negative bacteria. This bacteriocin also demonstrations powerful antilisterial activity in milk. Partially purified lactococcin BZ (400–2500 AU/ml) was demonstrated in a review to decrease *L*. *monocytogenes* count to an undetectable level in both skim and full‐fat milk during the storage at 4 and 20°C. In addition, this antilisterial activity was stable until the end of the storage period^25^ and was not adversely influenced by the milk fat content.^223^

**TABLE 8:** Bacteriocins of *Lactococcus* and their characteristics

Bacteriocin	Species and strain	Source	Mol. wt. (kDa)	Heat Stability range	Sensitivity	PH Stability range	Production phase	Optimal production	Inhibitory activity spectrum	Reference
BacL49	*L. lactis*	Moribund sleepy cod	5 and 54	At 100°C for 1 h	Proteinase K α‐chymotrypsin Trypsin Papain	2.5–9.5	Heart infusion broth (BHI) or agar (10–12 h)	Heat: 28°C PH: 5.5	*S. iniae*	224
LABW4	*L. lactis*	Naturally fermented milk	—	—	Proteinase K	5–7	MRS broth (24 h)	Heat:37°C PH: 6.5	*L*. *monocytogenes* MTCC657V	225
A5	*L. lactis* A5	Gastro‐intestinal tissues of Broadhead catfish	3.4	At 121°C for 15 min	Proteinase K	—	MRS broth (18 h)	Heat: 37°C	*B. cereus* *S. aureus* *S. thyphimurium*	226
Gt28, Gt29, and Ella8	*L. lactis*	Native fruits of Ecuador subtropical rainforest	—	At 121°C for 15 min	Proteinase K Pepsin Lysozyme α‐chymotrypsin	2–10	MRS broth (20 h)	Heat: 37°C PH: 6.0	*S*. *enterica* ATCC 51741 *E*. *coli* ATCC 25922	227
CJNU 3001	*L. lactis*	Kimchi	—	At 100°C for 15 min	Protease		MRS or RCM broth (24 h)	Heat: 25, 30, and 37°C	*S*. *aureus* KCTC 3881	228
Nisin Z‐producing	*lactis subsp. lactis KT2W2L*	Brackish water from a mangrove forest	—	At 100°C for 10 min	—	7	M17 medium (24 h)	Heat: 25°C PH: 7.0	*Brochothrix thermosphacta* DSMZ 20171T *S*. *aureus* CIP 76.25	229
Nisin Z	*L. lactis*	Freshwater fish	4.10	At 80°C for 15 min	Catalase Trypsin α‐Amylase Lipase	2–10	MRS broth (15 h)	Heat: 30°C PH: 6.5	*L*. *plantarum* 5S *E*. *faecalis* ATCC 51299 *S*. *aureus* ATCC 25923	230
BGBU1‐4	*L. lactis*	Cheese making	—	—		—	MRS broth (48 h)	Heat: 37°C	*L*.*monocytogenes* ATCC19111 *S*. *aureus* LMM322	231
Ent35‐MccV	*L. lactis* NZ9000	Milk	1.5–15	At 100°C for 10 min	Protease	7	M17 broth (16 h)	Heat: 30°C PH: 6.5	*L. monocytogenes* *E. coli*	232
LABW4	*L. lactis*	Home‐made buttermilk	‐	At 121°C for 15 min	Trypsin Papain Proteinase K Lysozyme	3–11	MRS broth (48 h)	Heat: 28°C PH: 5–7	*S*. *aureus* MTCC96 *P*.*aeruginosa* MTCC741	233
DF4Mi	*L. lactis*	Goat milk	‐	At 100°C for 2 h, and at 121°C for 20 min	Proteinase K Protease Pepsin α‐chymotrypsin	2–10	MRS broth (48 h)	Heat: 30°C PH: 5–7	*L. monocytogenes* ATCC 7644	234

### 11.2 Bacteriocins of Gram‐negative bacteria

Bacteriocins of Gram‐negative bacteria could be classified into three groups: peptide bacteriocins, colicin‐like bacteriocins (CLBs), and tailocins. Recent studies on *E*. *coli*, *S*. *enterica*, *Hafnia alvei*, *C*. *freundii*, *K*. *oxytoca*, *K*. *pneumoniae*, and *E*. *cloacae* have revealed that bacteriocin production level varies from 3% to 26% among environmental isolates.

#### 11.2.1 Colicin

Colicins produced by *E*. *coli* are present in 30–50% of strains isolated from human hosts. *E*. *coli* colicins have been the most widely studied Gram‐negative bacteriocins since their discovery; they are now used as a model system for studying bacteriocin structure/function, genetic organization, ecology, and evolution.^235^ Nomura and Witten^236^ showed that colicins E1 and K inhibited macromolecular synthesis without arrest of respiration, colicin E2 triggered DNA breakdown, and colicin E3 inhibited protein synthesis. According to recent evidence, the frequency of bacteriocin production in *E*. *coli* populations could vary from 10 to 80%, depending on the animal host from which they are isolated, the diet of the host, temporal changes, and the type of bacteriocin produced by the strain. When colicins enter the target cell, they could be divided into three categories based on their bactericidal mechanisms as follows: (i) pore‐forming colicins, including colicins A, B, E1, Ia, Ib, K, and N, are those colicins that act through the formation of pores or channels in the inner‐membrane to cause leakage of cytoplasmic compounds, destruction of electrochemical gradient, ion loss, and cell death; (ii) nuclease colicins, including colicins E2 to E9, are those colicins that contain DNase, 16S rRNase, and tRNase and act non‐specifically to digest cellular DNA and RNA of target bacteria; and (ii) peptidoglycanase colicins are proteins that could digest the peptidoglycan precursor, preventing the synthesis of peptidoglycan by bacteria and ultimately leading to bacterial death.^4^ Bacteriocins have been reported to inhibit important animal and plant pathogens, such as Shiga toxin‐producing *E*. *coli* (STEC), enterotoxigenic *E*. *coli* (ETEC), MRSA, VRE, *Agrobacterium*, and *Brenneria* spp.^7^ Studies have found that 20 different *E*. *coli* strains could produce colicin with inhibitory activity against five different Shiga toxin‐producing *E*. *coli* strains (O26, O111, O128, O145, and O157: H7). In humans, this *E*. *coli* strain (STEC) could cause diarrhea and hemolytic uremic syndrome. *E*. *coli* strains, producing colicins E1, E4, E8‐J, K, and S4, were shown in a study to significantly inhibit the growth of STEC in a simulated cattle rumen environment (…). Cutler et al.^237^ examined purified colicins E1 and N in vitro against enterotoxigenic *E*. *coli* F4 (K88) and *E*. *coli* F18, inducing post‐weaning diarrhea in piglets. In addition, purified colicin E1 protein was mixed into the diet of young pigs.

#### 11.2.2 Colicin‐like bacteriocins

CLBs (colicin‐like bacteriocins) target strains closely related to the producer bacteria usually of the same species and have species‐specific names; colicins, klebicins, and pesticins are derived from *E*. *coli*, *Klebsiella*, and *Yersinia pestis*, respectively. S‐type pyocins (named after the second part of the name *P*. *pyocyanea*) are formed by more than 70% of *Pseudomonas* strains. CLBs are modular proteins that are protease‐resistant and contain domains involved in receptor binding, translocation, and cytotoxicity.^238^

#### 11.2.3 Tailocins

Tailocins, also known as high molecular mass bacteriocins, are similar to the tail structures of *Myoviridae* and *Siphoviridae* bacteriophages. Most Gram‐negative and Gram‐positive bacteria have tailocin gene clusters in their genomes. The contractile tail of P2‐like temperate enterophages is morphologically and genetically similar to R‐type pyocins. Tailocins, unlike CLBs, are not protease‐resistant and are not classified as immunity proteins. The mechanisms of resistance to tailocins are unknown; however, it is thought to be mediated by a change in the cell surface receptors recognized by tailocins.^238^

#### 11.2.4 Microcin

In addition to colicins, *E*. *coli* strains could produce microcins, which are smaller than colicins and have more properties in common with bacteriocins produced by Gram‐positive bacteria, such as thermostability, protease resistance, relative hydrophobicity, and resistance to extreme pH.^239^ So far, 14 microcins have been discovered, but only seven have been isolated and thoroughly characterized. Microcins are low molecular weight (10 kDa), ribosomally synthesized, hydrophobic, antimicrobial peptides that differ from high molecular weight colicin proteins (25–80 kDa). Microcins are made up of N‐terminal leader and core peptides and are formed as precursor peptides. Microcins are primarily produced by *Enterobacteriaceae*, which are highly resistant to heat, pH, and protease. Microcins have a variety of bactericidal mechanisms, including pore formation, nuclease functions such as DNase and RNase, and inhibition of protein synthesis or DNA replication. No microcin gene cluster has a corresponding lysis gene; microcins are secreted outside the bacteria through the Type I ABC (ATP‐binding cassette) transporter secretion system, which is made up of a variety of proteins.^240^ According to their molecular masses, disulfide bonds in their structure, and post‐translational modifications, microcins are divided into two classes. Class I microcins, such as microcins B17, C7‐C51, D93, and J25, are post‐translationally modified peptides with a low molecular weight (less than 5 kDa). Class II microcins have a higher molecular weight (5–10 kDa) than Class I microcins. Class II microcins are further classified into two subclasses: Class IIa and Class IIb. To synthesize and assemble functional peptides, Class IIa microcins, such as microcins L, V, and N, require three separate genes. Microcins in class IIb, such as microcins E492, M, and H47, are linear peptides with/without C‐terminal post‐translational modifications.^241^ Cursino et al. (2006) examined simultaneous administration of probiont and enteric pathogen *S*. *flexneri* to germ‐free mice, which resulted in a strong inhibition of the pathogen. The synthesis of microcin C7 as well as colicins E1 and Ib was responsible for the observed inhibition. Inhibition of *S*. *flexneri* was mediated by microcin C7 production, while colicins E1 and Ib acted as a stabilization mechanism.^242^

### 11.3 New bacteriocin

Bacteriocins are ribosomally synthesized peptides produced by lactic acid bacteria (LAB), which have the potential to be used as food preservatives as well as antibiotics against multidrug‐resistant pathogens.^60^ The discovery of new bacteriocins with different properties indicates that there is still a lot of information to be understood about these peptide antibiotics. In this review, articles entitled with new bacteriocins from 2014 to 2020 were investigated (Table 9). Newly identified bacteriocins may differ in many respects, including the type of classes and subgroups to which they belong, biosynthetic mechanisms, structural characteristics, modes of antimicrobial action, and sources of isolation. Ahmad et al.^243^ investigated a new bacteriocin produced by *Lysinibacillus* with the potential to prevent the growth of foodborne bacterial and fungal pathogens, especially *B*. *pumilus* producing pumilacidins toxin. Given that 14 novel species of *Lysinibacillus* have been identified so far, none of which have been able to act against *B*. *pumilus*. It would be beneficial to look for new bacteriocin‐producing LAB in the gastrointestinal tracts of different marine animals because they have already been adapted to a seafood environment.^244^ Zhang et al.^49^ discovered PE‐ZYB1 as a new bacteriocin generated by *P*. *pentosaceus* zy‐B, isolated from Mimachlamys nobilis, with antimicrobial activity against *L*. *monocytogenes*. This bacteriocin could be used in the seafood industry as an important weapon against marine animal‐associated bacteria.^245^ Some bacteriocins have a high specific activity, making them a good candidate for use in clinical cases against multidrug‐resistant species because they could function at low concentrations with high specificity.^246^ They offer a possible solution to combat against MDR pathogens. Advances in science and technology to identify and purify bacteriocins make it possible to discover new bacteriocins that could control undesirable microorganisms (causing spoilage and pathogenesis).

**TABLE 9:** Characteristics of new bacteriocins

Bacteriocin	Species and strain	Source	Mol. wt. (kDa)	Heat stability range	pH Stability range	Stability	Sensitivity	Production phase	Inhibitory activity spectrum	Reference
PE‐ZYB1	Pediococcus pentosaceus zy‐B	Intestine of Mimachlamys nobilis	2.02 (LC‐MS/MS)	At 121°C for 20 min	2.0–7.0	Papain Pepsase	Trypsin	MRS (48 h)	*L. monocytogenes,* *S. hemolyticus* *S. aureus* *V. parahemolyticus,* *E. coli,* *E. aerogenes,* *P. aeruginosa* *B. subtilis* *B. cereus*	49
Bacteriocin SLG10	Lactobacillus plantarum SLG10	Kombucha	1.4 (MALDI‐TOF‐MS)	(−)	2.0–7.0	Trypsin Pepsin	Proteinase K Chymotrypsin Pepsin	MRS (30 h)	*B. subtilis* *B. cereus* *B. megaterium* *M. luteus* *B. thermosphacta* *C. butyricum* *S. aureus* *L.innocua* *L. monocytogenes* *E. coli*	247
Bacteriocin SF1	S. flexneri serotype 2a	Dysenteric diarrhea	66 (glycine SDS‐PAGE)	At 76–80°C for 30 min	2 to 7	Trypsin *α*‐chymotrypsin Pepsin proteinase K papain	Proteinase K Papain	BHI (24 h)	*E. coli* *B. fragilis*	248
Un name	Lysinibacillus JX402121	Spoiled fruits and vegetable waste	51 (SDS‐PAGE)	up to 80°C temperature	4 to 9	Metal ion Amylase Lipase Surfactants Solvents	Proteinase K Pepsin Trypsin	MRS (48 h) aerobically	*B. pumilus* *B. pumilus* *B. subtilis* *S. epidermidis* *S. aureus* *L. monocytogenes* *M. luteus* *B. cereus* *P. aeruginosa* *A. niger* *A. fumigatus* *F.oxysporium* *A. flavus* *T. viridae*	243
BacBS2	Bacillus velezensis BS2	Meongge Jeotgal	6.5 (Tricine‐SDS PAGE)	At 80°C for 15 min	4–9	Pepsin Trypsin	Protease Proteinase K	LB	*L. monocytogenes* *B. cereus*	249
Subtilin L‐Q11	*Bacillus subtilis* L‐Q11	Orchard Soil	3.6 (MALDI‐TOF‐MS)	At 60°C for 15 min or 80°C for 30 min	2 to 7	Detergents	Trypsin Chymotrypsin Proteinase K Pepsin	LB	*B.amyloliquefaciens* *L. lactis* *L. plantarum* *S. aureus* *E. faecalis* *S. aureus ATCC 29213* *Bacillus spp*	250
Bacteriocin CAMT2	*Bacillus amyloliquefaciens* ZJHD3‐06	*Epinephelus areolatus*	(−)	(−)	(−)	(−)	(−)	Modified tryptone glucose extract	*L*. *monocytogenes* ATCC 19111	251
Virgicin	*Virgibacillus* sp. strain AK90	Saltpan	2.4 (MALDI analysis)	At 100°C for 15 min	4 to 8	(−)	Trypsin Proteinase K	NB (24 h) Logarithmic phase	*M. luteus MTCC 106* *S. aureusMTCC 1430* *S. aureus MTCC 96* *B. subtilis MTCC 121* biofilm formation of *Enterococcus faecalis*	252
Gassericin S	*L. gasseri LA327*	Large intestine tissue in humans	5.4	At 121°C for 15 min	2–10	(−)	Proteinase K Trypsin a‐chymotrypsin	MRS	(−)	253
Plantaricin LPL−1	*Lactobacillus plantarum LPL−1*	Fermented fish	4.4 (MALDI‐TOF‐MS)	At 121∘C for 20 min	2–10	EDTA Tween 20 Tween 80 Urea	Pepsin Papain Proteinase K trypsin Chymotrypsin	MRS broth for 32 h at 37°C (exponential growth phase at 4 h)	*S. aureus* *L. monocytogenes* *B. pumilus* *B. amyloliquefaciens* *E. faecalis* *L. plantarum* *L. delbrueckii, Lb. bulgaricus* *L. Salivarius,* *L. lactis*	48
Bacteriocin DY4‐2	*L. plantarum*	Cutlass fish	1.5 (MALDI‐TOF‐MS)	At 121 °C for 30 min	2.5–5.5	(−)	Pepsin Trypsin Papain	MRS broth at stationary phase after 24 h incubation	Gram‐negative bacteria: *P. fluorescens P001* *P. fluorescens PF−04* *L. monocytogenes19115* *P. aeruginosa 9027* *Shewanella putrefaciens 8071* *V. harveyi BB107* *Psychrobacter sp. 00052* Gram‐positive bacteria: *Bacillus cereus 63301 B.licheniformis 11091*	254
Plantaricin JY22	*L.plantarum JY22*	Golden carp intestine	4.1	At 121 °C for 20 min	2.5 to 5.5	Amylase	Nutrase Papain Pepsin	MRS (24 h)	*Bacillus cereus*	255
Plantaricin ZJ316	*L.plantarum ZJ316*		2.3	At 121 °C for 30 min	2.0 to 10.0		α‐chymotrypsin Trypsin Proteinase K	MRS	*S.aureus* *E. faecalis* *M.luteus* *B. subtilis* *P. aeruginosa* *P. putida* *E. coli* *Acetobacter aceti,* *Salmonella enterica* *Vibrio parahaemolyticus* *L. monocytogenest* *Listeria welshimeri*	256
Plantaricin GZ1‐27	*L. plantarum WCFS1*	Kipper of Dong	0.97 (MALDI‐TOF/MS)	At 80°C for 30 min	2.0–6.0	(−)	Proteinase K Papain Trypsin Pepsin	MRS (36 h)	*Spoilage bacteria:* *B. thermosphacta* *P. fluorescens* *A. baumannii* *Pathogenic bacteria:* *B. cereus* *S. aureus* *S. typhimurium* *L. monocytogenes* *E. coli*	257
Bifidoadocin	*B. adolescentis*	(−)	14.6	At 90 °C for 15 min	3–8	(−)	Chymotrypsin Pepsin Papain	(−)	*K.pneumoniae* *A. baumanii* *Staphylococcus spp*. *Salmonella spp*. *P. aeruginosa* *A.hydrophila*	210
Fermencin SA715	*Lactobacillus fermentum GA715*	Goat milk origin	1.8 (MALDI‐TOF)	At 40–90°C for 30 min	2–7	Lysozyme Lyticase Catalase	Proteinase K Peptidase Trypsin α‐chymotrypsin Protease	MRS (18 h)	*M.s luteus ATCC 10240* *Corynebacterium spp. GH17* *P.aeruginosa PA7* *S. aureus RF122* *L. monocytogenes NCTC10890* *S. mutans GEJ11* *S. equisimilis ATCC 12388,* *S. sanguinis ATCC 10556* *B.cereus ATCC 14579* *E.coli UT181*	258
Salivaricin mmaye1	*Lactobacillus salivarius SPW1*	Human feces	1.2 (MALDI‐TOF)	At 121°C for 15 min	2–10	Detergents Protease	Pepsin Trypsin α‐chymotrypsin Protease Proteinase	MRS (18 h)	*Streptococcus* *Lactococcus* *Listeria* *Staphylococcus* *Corynebacterium* *Pseudomonas* *Escherichia* *Micrococci* *Lactobacillus* *Enterococci*	259
Pentocin MQ1	*Lactobacillus pentosus CS2*	Coconut	2.1 (MALDI‐TOF)	At 121°C for 15 min	2–5	Detergents Protease	Proteinase K Pepsin Proteinase Trypsin α‐chymotrypsin Protease	MRS broth at 37°C for 20 h	*L. monocytogenes NCTC 10890* *M. luteus ATCC 10240* *B. cereus ATCC 14579* *S. pyogenes ATCC 12344* *S. aureus RF122* *P. aeruginosa PA7* *E. faecium ATCC 19434* *E. faecium ATCC 27270* *E. faecium ATCC 27273* *E. faecium ATCC BAA‐2318* *E. faecium ATCC BAA‐2127* *E. faecium ATCC 6569* *E. faecium ATCC 25307* *E. faecium ATCC 349*	260
Plantaricyclin A (PlcA)	*Lactobacillus plantarum NI326*	Olives	5.5 (MALDI‐TOF)	At 100°C for 10 min	2–10	Proteases	Proteinase K Pronase	MRS broth supplemented with 0.8% bacteriological agar	*A. acidoterrestris sp1* *Lb. bulgaricus UCC* *Pediococcus inopinatus 1011 Lactococcus lactis HP UCC* *L. lactis KH UCC* *L. lactis MG1363 UCC* *L. lactis RT28 UCC* *L. lactis NZ9000*	261
Bacteriocin SKI19	*Lactobacillus plantarum subsp. plantarum SKI19*	Sai krok e‐san mu”	2.5	At 121°C for 15 min	2–10	(−)	α‐chymotrypsin Trypsin Proteinase K Pronase E	MRS (24 h) early stationary phase	*L. monocytogenes DMST 17303* *E.coli DMST 4212* *S. aureus DMST 8840*	262
Leucocin C−607, 607A	*Leuconostoc pseudomesenteroides 607*	Persimmon fruit	C−607 (4.6) 607A (3 and 3.1)	At 121°C for 15 min (C−607) At 80/90°C for 30 min (607A)	(−)	(−)	Proteinase K Trypsin	MRS (72 h)	Multidrug‐resistant *S*. *aureus* *L. casei ATCC 393* *MRSA GIM 1.771* *S. dysenteriae CGMCC 1.1869* *E. coli O157:H7 GIM 1.707* *B. subtilis CGMCC 1.1627* *E. faecalis ATCC 51575,* *L.monocytogenes ATCC 19112* *M. luteus CGMCC 1.2299* *P. aeruginosa CGMCC 1.1785*	263
Plantaricin DL3	*Lactobacillus plantarum DL3*	Chinese Suan‐Tsai	4.1	At 121°C for 15 min	2.5–5.5	α‐‐amylase	Pepsin Trypsin Papain	MRS (24 h)	*P*. *aeruginosa*. *L. monocytogenes* *P. aeruginosa* *S. putrefaciens* *Psychrobacter sp*. *Gram‐positive* *S. aureus* *B. cereus* *B.licheniformis* *P. fluorescens,* *S. putrefaciens*	264
Mejucin	*Bacillus subtilis SN7*	Meju	(−)	(−)	(−)	Enzymes (α‐amylase or lipase) solvent (Ethanol Acetonitrile, Acetone, Methanol)	Proteinase K Protease α‐Chymotrypsin Pepsin Aminopeptidase I Carboxypeptidase Mercaptoethanol	TSB at 37 C for 24 h	*B. cereus strains* *L.monocytogenes* *S. aureus ATCC 29213* *M. luteus ATCC 15307*	265

## 12 THE PROBIOTIC APPLICATION OF BACTERIOCINS

### 12.1 The GI tract

The human gastrointestinal tract is a complex environment in which the intestinal microflora and the host maintain a delicate balance. The microflora acts as a primary stimulus for the development of the mucosal immune system. The two main genera of lactic acid bacteria, including *Lactobacillus* and *Bifidobacterium*, dominate the intestinal flora with 56 *Lactobacillus* and various *Bifidobacterium* species. In in vitro, the majority of these organisms have been shown to produce bacteriocins. A compelling in vivo analysis demonstrated the activity of *Ligilactobacillus salivarius* strain UCC118 that produced a potent broad‐spectrum bacteriocin (Abp118) active against the foodborne pathogen *L*. *monocytogenes*.^266^ In a study, an enterohemorrhagic strain of *E*. *coli* and a strain of *L*. *monocytogenes* were shown to significantly inhibit an enterohemorrhagic strain of *E*. *coli* and a strain of *L*. *monocytogenes* in mice, most likely due to bacteriocin activity.^267^ The release of bacteriocins inhibiting *H*. *pylori*, as a human pathogen that causes severe gastroduodenal diseases, has been chiefly studied in *lactobacilli* strains. Anti‐*H*. *pylori* activity of probiotic strains *L*. *johnsonii* LA1 and *L*. *acidophilus* LB was identified by Gillor et al. (2008). According to the results, when *H*. *pylori* was attached to intestinal epithelial cells in both cases, inhibitory activity was retained. Also, oral administration of *L*. *acidophilus* LB in mice protected the animals from *H*. *felis* infection. This probiotic bacterium (LB) was also shown to inhibit gastric colonization and prevent gastric inflammation. Oral intake of live bacteria by *H*. *pylori*‐positive school children resulted in a significant decrease in urease production, while administration of *L*. *johnsonii* LA1 supernatant to adult patients colonized by *H*. *pylori* significantly decreased infection.^268^

### 12.2 Treatment of pathogen‐associated diseases

Stern et al. (2006) purified Class II low molecule mass bacteriocin OR‐7 from *L*. *salivarius* strain NRRL B‐30514, which was potent in inhibiting *C*. *jejuni* causing human gastroenteritis. OR‐7 was resistant to lysozyme and lipase therapy, heat up to 90°C, and pH levels of 3.0 to 9.1. For chicken feed, purified OR‐7 was encapsulated in polyvinylpyrrolidone. In the cecal material of OR‐7‐treated chickens, the population of *C*. *jejuni* decreased by at least one million fold compared with chickens not given OR‐7 supplementation. These results suggest the high potential of nisin, OR‐7, and other bacteriocins for use as suitable alternatives to antibiotics in poultry and other animal feeds.^269^ Only a few bacteriocins have been tested against *C*. *difficile* infection so far (CDI). The mode of delivery of bacteriocins to the colon must be carefully analyzed when using bacteriocins as an alternative/adjunctive therapeutic choice for CDI. Encapsulation of bacteriocins could be a way to avoid the protease effect.

#### 12.2.1 Thuricin CD

Thuricin CD is a recently identified bacteriocin with effective narrow‐spectrum inhibitory activity against *C*. *difficile*. The primary advantage of thuricin CD is that its antimicrobial activity is mostly restricted to *C*. *difficile* and has little or no impact on other gut microorganisms. This was demonstrated in a study using a human distal colon model and a high‐throughput sequencing approach, which revealed that thuricin CD had minimal impact on the numbers of Firmicutes, Bacteroidetes, and Proteobacteria compared with vancomycin and metronidazole, eliciting a decrease in Firmicutes and Bacteroidetes numbers as well as an increase in Proteobacteria number.^270^

#### 12.2.2 Lacticin 3147

Lacticin 3147 is a 2‐peptide antibiotic produced by *L*. *lactis* DPC 3147. Lacticin 3147 was shown by Rea et al. (2007) to trigger fast lysis of log‐phase *C*. *difficile* cells, measured by quantifying the release of acetate kinase. The addition of lacticin 3147 at a high concentration of 6 mg/ml resulted in a decrease in *C*. *difficile* ATCC 43593 cell number from 106 cfu/ml to zero within 2 h. Subsequent studies displayed that the use of lacticin 3147 resulted in a decrease and increase in Firmicutes and Proteobacteria numbers, respectively.^271^ Actagardine A is a 19‐amino acid lantibiotic with powerful antimicrobial activity against Gram‐positive bacteria, including *C*. *difficile*. NVB302 is a semi‐synthetic Type B lantibiotic extracted from actagardine, which is effective against *C*. *difficile* and performs better than vancomycin.^59^

#### 12.2.3 LFF571 (GE2270 derivative)

GE2270 is a thiopeptide bacteriocin that inhibits translation in bacteria. LFF571 is a semi‐synthetic derivative of thiopeptide GE2270, developed by Novartis Co., which displays antimicrobial activity against a range of Gram‐positive bacteria, including *C*. *difficile*. In vivo human activity of LFF571 against *C*. *difficile* was compared with vancomycin in a survey on gold Syrian hamster models. Only 2.2% of hamsters experienced recurrence in the case of treatment with 5 mg/kg of LFF571, whereas 37.8% of hamsters which survived at the termination of treatment with 20 mg/kg of vancomycin experienced recurrence.^272^ Durancin 61A as a glycosylated bacteriocin produced by *E*. *durans* 61A was investigated in a study by Hanchi et al. (2017) and shown to be active against clinical drug‐resistant *C*. *difficile*, *E*. *faecium*, VRE, and MRSA. In their study, durancin 61A exhibited bactericidal activity and acted on the bacterial membrane with a pore‐formation mechanism, including pore and vesicle formation, cell disruption, and loss of membrane integrity and cytoplasm content. The observed increase in durancin 61A MIC for nisin‐ and pediocin‐resistant variants could be probably due to their similar mode of action on bacterial membrane. The absence of cross‐resistance in durancin‐resistant variant of *E*. *faecalis* ATCC 27275 suggests a different site of action for this bacteriocin. The susceptibility of vancomycin‐resistant variants to durancin 61A could be due to the altered cell wall physiology and metabolism of vancomycin‐resistant variants.^273^

#### 12.2.4 Pediocin PA‐1

One of the main subclasses of bacteriocins is the pediocin family, a title derived from pediocin PA‐1 as the first and extensively studied member of this family. The pediocin family members are small, heat‐stable, membrane‐active peptides that contain no lanthionine and possess a YGNGVXC consensus motif. Members of this class have several features in common, considering a very strong antimicrobial activity against *L*. *sakacin P* and *L*. *sakacin G*.^274^ It has been shown that *L*. *curvatus* FS 47 produces curvaticin FS47 active against *L*. *monocytogenes*. Besides the strain *L*. *plantarum* C11 as the first strain isolated from a plant source, four other strains of *L*. *plantarum*, including WCFS1, NC8, 51, and J23, have been found to harbor YGNGVQCilar bacteriocin locus in their genomes, peculiarly plantaricin A (PlnA) as a cationic peptide pheromone with membrane permeabilizing activity.^275^ The 2‐element Class II bacteriocins derived from *L*. *salivarius* isolated from mammals have been characterized as follows: ABP‐118 (produced by human intestinal probiotic strain *L*. *salivarius* UCC118), L. salivaricin CRL 1328 (produced by *L*. *salivarius* CRL 1328 strain isolated from the healthy human vagina), and salivaricin P (produced by porcine intestinal probiotic strain *L*. *salivarius* DPC6005).^276^ FK22, OR7, L‐1077, and SMXD51 bacteriocins derived from *L*. *salivarius* isolated from chicken intestines or intestinal contents (L. salivarius K7, L. salivarius NRRL B‐30514, L. salivarius 1077 NRRL B‐50053, and L. salivarius SMXD51) have been defined as one‐factor Class II bacteriocins.^277^ ABP‐118 is an appealing broad‐spectrum antimicrobial substance since it could inhibit several foodborne and medically notable pathogens, such as *Bacillus*, *Listeria*, *Enterococcus*, and *Staphylococcus* genera, without a clear antagonistic activity toward related LAB. ABP‐118 is a Class IIb two‐peptide bacteriocin consisting of Abp118α and Abp118β. The Abp118α peptide possesses inhibitory activity when concentrated, and the presence of the Abp118β peptide enhances this activity; ABP‐118 does not have the related bacteriocin activity on itself.^278^ LS1 is a human bacteriocin, produced by *L*. *salivarius* BGHO1 as a human oral isolate, with antagonistic activity against *S*. *mutans*, *S*. *pneumoniae*, *S*. *aureus*, *E*. *faecalis*, *M*. *flavus*, and *S*. *enteritides*.^276^ Wu et al.^251^ conducted a study to investigate the mode of action of bacteriocin CAMT2, produced by *B*. *amyloliquefaciens* ZJHD3‐06, against highly pathogenic *L*. *monocytogenes* ATCC 19111; it exhibited inhibitory activity against important food spoilage and foodborne pathogens. As a member of acyldepsipeptide antibiotics, ADEP‐1 activity is through interrupting protein metabolism in the bacterial cell by eliciting over‐activation of intracellular caseinolytic ATP‐dependent proteases. Therefore, interruption of intracellular protein metabolism leads to cell division, differentiation, sporulation impairment, and finally bacterial cell death. Interestingly, it has been shown that CD proteases targeted by acyldepsipeptide antimicrobials are also related to the intracellular systems sigma factors σ (E) and MazEF, which play a key role in CD sporulation. Given the bactericidal activity of ADEP‐1 against Gram‐positive bacteria and caseinolytic proteases in CD, as the potential target of this compound, some authors have recently proposed the use of ADEP‐1 and other acyldepsipeptide antimicrobials to treat CDI, either alone or in combination with other antimicrobial classes.^279^

## 13 FOOD TECHNOLOGY

In food technology, nisin as the first antibacterial peptide found in LAB is produced by *L*. *lactis*. It is also a commercial bacteriocin marketed as Nisaplin^®^, which is used as a food preservative against contamination with microorganisms. It is the only bacteriocin approved by USFDA for use as a preservative in many food products and licensed as a food additive in over 45 countries. Another commercially available bacteriocin is pediocin PA‐1 marketed as Alta^®^ 2341, which inhibits the growth of *L*. *monocytogenes* in meat products. Enterocin AS‐48 is used in cider, fruit and vegetable juices, and canned vegetables for contamination inhibition. Enterocins CCM4231 and EJ97 are used in soy milk and zucchini purée to suppress contamination, respectively.^280^ The use of bacteriocins as food preservatives may be beneficial in several aspects: (i) to decrease the risk of food poisoning, (ii) to decrease cross‐contamination in the food chain, (iii) to improve the shelf life of food products, (iv) to protect food during temperature‐abuse episodes, (v) to decrease economic losses due to food spoilage, (vi) to reduce the level of chemical preservatives added, (vii) to reduce the intensity of physical treatments in order to better preserve food nutritional value and decrease processing costs, and (viii) to provide alternative preservation methods for "novel food" in order to meet the demands of consumers for fresh‐tasting, lightly preserved, ready‐to‐eat food products.^281^

## 14 CONCLUSION

Given their broad‐spectrum and potent inhibitory activity, this study results indicated that lactic acid bacteria are able to exert antagonistic activity against pathogenic bacteria *in vitro* and *in vivo*. This study provides a comprehensive overview of what is known about LAB bacteriocins, especially their properties, classification, biology, sources, biosynthesis, potential applications, activity against foodborne pathogens, impact on antibiotic‐resistant bacteria, mode of action, genetic determinants, range of activity, and factors affecting their production. Many strains of lactic acid bacteria related to food groups could produce bacteriocins or antibacterial proteins that are highly effective against foodborne pathogens, such as *S*. *aureus*, *P*. *fluorescens*, *P*. *aeruginosa*, *S*. *typhi*, *S*. *flexneri*, *L*. *monocytogenes*, *E*. *coli O157*:*H7*, *K*. *pneumoniae*, and *C*. *botulinum*. A wide range of bacteria belonging primarily to the genera Bifidobacterium and Lactobacillus, even some gram‐negative bacteria like *E*. *coli*, have been characterized with different health‐promoting attributes. The frequency and variety of bacteriocin production vary greatly between populations of gram‐positive and gram‐negative bacteria. Extensive and in‐depth studies have been performed on gram‐positive bacteria and also relatively few on gram‐negative bacteria. Production of two or more bacteriocins by one cell in gram‐negative bacteria is a common phenomenon, at least in *E*. *coli*. More research is needed to understand the nature of the fitness benefits of producing multiple bacteriocins and how to optimally use bacteriocins in gram‐negative bacteria as alternatives to traditional antibiotics and to create and select bacterial strains for use as probiotics.

Studies results describe bacteriocins as supplements for antibiotics due to their high stability, no side effects, and potential to induce a synergistic effect. In combination with antibiotics, bacteriocins act synergistically; they not only increase the efficiency of antibiotics and prevent the emergence of antibiotic‐resistant species but also reduce the side effects of antibiotics by lowering the concentration of antibiotics needed to eliminate bacteria. The results of recent studies on this topic show that bacteriocin as being a versatile antimicrobial with appreciable potency for utilization as biopreservatives, antibiotic alternatives, health‐promoting gut modulators, and animal growth promoters. One of the reasons for the positive trend toward bacteriocins is the overuse of antibiotics, especially broad‐spectrum antibiotics in medicine and food production, which is known as a microbiome disorder and for the accumulation and transfer of resistance genes in the intestinal microbial population. Man has been chosen.

According to the present study results, the correct selection of bacteriocin‐producing strains suitable for use in the food industry is very important. Thus, this emphasizes the necessity of investigating LAB bacteriocins to prove their beneficial and nutritional properties as well as inhibitory activity against the growth of functional pathogens, as they are potentially crucial for the final preservation of functional foods and for medicinal applications.

According to the present study results, the correct selection of bacteriocin‐producing strains suitable for use in the food industry is very important. Thus, this emphasizes the necessity of investigating LAB bacteriocins to prove their beneficial and nutritional properties as well as inhibitory activity against the growth of functional pathogens, as they are potentially crucial for the final preservation of functional foods and for medicinal applications.

## CONFLICT OF INTEREST

The authors declare that there is no conflict of interest.

## AUTHOR CONTRIBUTIONS

RG and MK conceived, designed, and supervised the study. AD and AA contributed to data collection, interpretation, and final approval of data for the work. AD and MM developed the first and final draft of the manuscript. EO and MT developed the second draft of the manuscript. ADE and MH designed and checked all figures and tables. All authors reviewed, contributed to the revisions, and finalized the drafts.

## Data Availability

All relevant data are included in the manuscript.
